# Plasminogen deficiency suppresses pancreatic ductal adenocarcinoma disease progression

**DOI:** 10.1002/1878-0261.13552

**Published:** 2023-11-27

**Authors:** Nayela N. Chowdhury, Yi Yang, Ananya Dutta, Michelle Luo, Zimu Wei, Sara R. Abrahams, Alexey S. Revenko, Fenil Shah, Lindsey A. Miles, Robert J. Parmer, Bas de Laat, Alisa S. Wolberg, James P. Luyendyk, Melissa L. Fishel, Matthew J. Flick

**Affiliations:** ^1^ Department of Pediatrics and Herman B Wells Center for Pediatric Research Indianapolis IN USA; ^2^ Indiana University Simon Comprehensive Cancer Center Indianapolis IN USA; ^3^ Department of Pharmacology and Toxicology Indiana University School of Medicine Indianapolis IN USA; ^4^ Department of Pathology and Laboratory Medicine University of North Carolina at Chapel Hill NC USA; ^5^ Lineberger Comprehensive Cancer Center University of North Carolina at Chapel Hill NC USA; ^6^ UNC Blood Research Center University of North Carolina at Chapel Hill NC USA; ^7^ Department of Pathobiology & Diagnostic Investigation Michigan State University East Lansing MI USA; ^8^ Institute for Integrative Toxicology Michigan State University East Lansing MI USA; ^9^ Department of Pharmacology and Toxicology Michigan State University East Lansing MI USA; ^10^ Ionis Pharmaceuticals Carlsbad CA USA; ^11^ Department of Molecular Medicine Scripps Research Institute La Jolla CA USA; ^12^ Department of Medicine, Veterans Administration San Diego Healthcare System University of California, San Diego CA USA; ^13^ Synapse Research Institute Maastricht The Netherlands

**Keywords:** metastasis, mouse model, PDAC, plasminogen

## Abstract

Pancreatic ductal adenocarcinoma (PDAC) is a highly fatal metastatic disease associated with robust activation of the coagulation and fibrinolytic systems. However, the potential contribution of the primary fibrinolytic protease plasminogen to PDAC disease progression has remained largely undefined. Mice bearing C57Bl/6‐derived KPC (*KRas* ^*G12D*^, *TRP53* ^*R172H*^) tumors displayed evidence of plasmin activity in the form of high plasmin–antiplasmin complexes and high plasmin generation potential relative to mice without tumors. Notably, plasminogen‐deficient mice (Plg^‐^) had significantly diminished KPC tumor growth in subcutaneous and orthotopic implantation models. Moreover, the metastatic potential of KPC cells was significantly diminished in Plg^‐^ mice, which was linked to reduced early adhesion and/or survival of KPC tumor cells. The reduction in primary orthotopic KPC tumor growth in Plg^‐^ mice was associated with increased apoptosis, reduced accumulation of pro‐tumor immune cells, and increased local proinflammatory cytokine production. Elimination of fibrin(ogen), the primary proteolytic target of plasmin, did not alter KPC primary tumor growth and resulted in only a modest reduction in metastatic potential. In contrast, deficiencies in the plasminogen receptors Plg‐RKT or S100A10 in tumor cells significantly reduced tumor growth. Plg‐RKT reduction in tumor cells, but not reduced S100A10, suppressed metastatic potential in a manner that mimicked plasminogen deficiency. Finally, tumor growth was also reduced in NSG mice subcutaneously or orthotopically implanted with patient‐derived PDAC tumor cells in which circulating plasminogen was pharmacologically reduced. Collectively, these studies suggest that plasminogen promotes PDAC tumor growth and metastatic potential, in part through engaging plasminogen receptors on tumor cells.

## Graphical abstract

PDAC tumor cells promote plasmin generation in the tumor microenvironment. Plasmin engages tumor cell receptors Plgrkt and S100A10:annexin A2 to enhance tumor growth and metastasis.

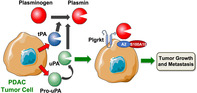

**Abbreviations**
AIF‐1: allograft inhibitory factor‐1
ASO: antisense oligonucleotide
EMT: epithelial‐to‐mesenchymal transition
EPP: endogenous plasmin potential
FDP: fibrin degradation products
G‐MDSCs: granulocytic myeloid‐derived suppressor cells
KPC: *KRas* ^*G12D*^, *TRP53* ^*R172H*^, *Elastase‐Cre* ^*ER*^
LLC: Lewis Lung Carcinoma
PAP: plasmin‐antiplasmin complexes
PAR: protease‐activated receptor
Plg: plasminogen
Plg‐RKT: plasminogen receptor‐KT
PPP: platelet poor plasma
ROI: regions of interest
TF: tissue factor
tPA: tissue plasminogen activator
uPA: urokinase plasminogen activator
uPAR: urokinase plasminogen activator receptor
VTE: venous thromboembolism

## 1 Introduction

Pancreatic Ductal Adenocarcinoma (PDAC) has the highest rate of mortality among all solid tumors with a 5‐year survival rate at diagnosis of 12%, and this decreases to 3% in metastatic disease according to the American Cancer Society [1]. It is one of the eight deadliest cancers in the United States with > 62 000 new cases expected to be diagnosed annually resulting in > 50 000 deaths. A common pathological manifestation in PDAC is local and systemic activation of the coagulation system. PDAC has the highest rate of cancer‐associated venous thromboembolism (VTE) and VTE is decidedly correlated with disease aggressiveness [2, 3, 4]. Notably, there is a reciprocal relationship between the coagulation system and PDAC as coagulation system components, such as tissue factor and prothrombin, have been linked to PDAC disease progression [5]. The most common type of oncogenic mutation in PDAC is an activating KRAS mutation, which is observed in > 90% of patient biopsies [6, 7]. Activated KRAS results in significant upregulation in expression of tissue factor (TF; *F3*) [8, 9, 10], the initiator of the coagulation cascade [11]. TF leads to activation of the central coagulation protease thrombin. Thrombin cleaves soluble fibrinogen leading to the formation of fibrin matrices. Thrombin also drives cell signaling through protease‐activated receptors (PAR)‐1, ‐3, and ‐4. TF, thrombin, and PAR‐1 have each been linked to PDAC tumor progression, and fibrin(ogen) has been implicated in supporting tumor growth and metastasis of multiple epithelial cell‐derived malignancies [5, 12, 13].

A counterbalance to the coagulation system is the plasminogen activation/fibrinolytic system. Plasminogen (Plg) is a 92 kDA single‐chain glycoprotein synthesized by the liver and is secreted in blood as an inactive zymogen [14]. Plasminogen is converted to its active form plasmin through the action of two plasminogen activators, tissue plasminogen activator (tPA) and urokinase plasminogen activator (uPA) [15, 16]. The classical role of plasmin is in fibrinolysis, which is the process of clearing fibrin‐rich matrices either in the form of blood clots or in the extravascular space. Notably, PDAC disease progression is also linked to upregulation in fibrinolytic proteins. Specifically, oncogenic KRAS upregulates uPA and uPA receptor (uPAR) expression in PDAC tumor cells, and uPA and uPAR expression in PDAC is strongly correlated with poor patient prognosis [17, 18, 19]. Increased levels of tPA have also been identified in PDAC tumor homogenates [20]. Current data suggest that pericellular proteolysis in the TME and tumor cell signaling via the uPA/uPAR system plays a crucial role in enabling tumor cell invasion, migration, and metastasis in pancreatic cancer [21, 22]. However, the potential contribution of plasminogen itself to PDAC tumor growth and metastasis has not been defined.

Here, we determine the role of plasminogen in PDAC tumor growth and metastasis using mouse and human xenograft models of pancreatic cancer. We demonstrate the efficacy of suppressing plasminogen in circulation and its effect on reducing tumor progression and metastatic burden. Elimination of plasminogen from the PDAC TME resulted in changes in the accumulation of tumor‐supporting cells and tumor cell survival. Finally, we investigated whether PDAC tumor growth was linked to the primary substrate of plasmin proteolytic activity and the expression of plasminogen receptors, including Plg‐RKT and S100A10 by tumor cells.

## 2 Materials and methods

### 2.1 PDAC cell lines

C57Bl/6J‐derived KPC2 tumor cell lines were generated from individual primary tumors derived from KPC (*Kras* ^G12D/+^, *p53* ^*R172H/+*^, *Elas‐Cre* ^*ER/+*^) mice at Purdue University as previously described [5]. KPC2‐GFP cells were previously described [5]. Cells were maintained in RPMI1640 medium containing 10% FBS and 1% penicillin/streptomycin. Lentivirus encoding shPlgrkt, shS100A10, and shControl were obtained from Sigma, Burlington, MA, USA (Plgrkt: TRCN0000305338, S100A10: TRCN0000097667, and Control: shRNA Control Transduction Particles SHC002V). KPC cell lines were pathogen tested by IDEXX Laboratories. The results of pathogen testing, including mycoplasma analysis, were negative. Low‐passage patient‐derived Pa02C and Pa03C cells were gifted from Dr. Anirban Maitra (Johns Hopkins University) and cultured in DMEM supplemented with 10% FBS. These human PDAC cells, Pa02C and Pa03C, were generated from hepatic metastatic lesions of male patients with stage IV pancreatic cancer. The Pa02C (RRID:CVCL_E302) and Pa03C (RRID:CVCL_E301) cells have KRAS Missense Q61H and G12D mutations and p53 Missense L257P and L344P mutations, respectively. STTR analysis was performed to authenticate these cells and routinely confirmed that they were mycoplasma‐free.

### 2.2 Subcutaneous and orthotopic tumor growth and experimental lung metastasis assay

All *in vivo* animal studies were conducted in compliance with guidelines of National Institutes of Health and approved by the Institutional Animal Care and Use Committees of the University of North Carolina at Chapel Hill (IACUC Protocol #22‐164.0) or the Indiana University School of Medicine (IACUC Protocol #21165). Experiments with KPC2 cells employed previously described plasminogen‐deficient mice [23], Plg‐RKT‐deficient mice [24], fibrinogen‐deficient mice [25], or fibrinogen^AEK^ mice on a C57Bl/6 background [26] as well as matched littermate controls were bred in‐house. The Plg‐RKT were generated at Scripps Research Institute but utilized at University of North Carolina at Chapel Hill. The plasminogen‐deficient, fibrinogen‐deficient, and fibrinogen^AEK^ mice have been bred in Flick laboratory since being generated. For each experiment, 8–12 week old male and female mice were analyzed in separate independent experiments and no difference in tumor growth and metastasis was detected based on sex. Mice were housed in 12‐h light/dark cycle with *ad libitum* access to food and water. For subcutaneous KPC2 tumor studies, cells were injected into the intrascapular region at a concentration of 2.5 × 10^5^ cells in 100 μL sterile PBS. Tumors were measured over time and tumor volume was calculated as: Volume = (Length × Width^2^)/2. At the end of the study, tumors and whole blood were harvested for analysis 4 weeks after injection. Orthotopic KPC2 cell injections were performed at a concentration of 5 × 10^4^ cells in 20 μL sterile PBS. Tumor tissue and whole blood were harvested 3 weeks after injection. For experimental metastasis assays, 5 × 10^4^ KPC2 cells or 2.5 × 10^5^ KPC2‐GFP cells in 200 μL PBS were administered by tail vein injection. Lung tissue was harvested at various time points, weighed, and fixed for histology. In KPC2 experiments in which circulating plasminogen was reduced pharmacologically, Plg antisense oligonucleotide (ASO) or control ASO was used as previously described [27]. Here, C57Bl/6 mice (University of North Carolina at Chapel Hill) received twice per week intraperitoneal injections with either Control ASO or Plg Gal‐Nac ASO at 7.5 mg/kg, as described [27, 28]. To ensure plasminogen levels were stably reduced at the time of tumor growth initiation, ASO treatment began 2 weeks prior to tumor cell injection. Foci were enumerated by counting total surface macrometastatic foci or counting GFP^+^ micrometastatic foci in the left lung lobe with imagej, as described [5].

Experiments evaluating human PDAC lines, Pa02C or Pa03C utilized NSG (NOD.Cg‐Prkdcscid Il2rgtm1Wjl/SzJ) male mice of 6–8 weeks of age purchased from the Preclinical Modeling and Therapeutics Core (IU Simon Comprehensive Cancer Center) and maintained in pathogen‐free conditions and a 12‐h light/dark cycle with *ad libitum* access to food and water. All food, water, bedding, and cages were autoclaved and mice were handled under a laminar flow hood. NSG mice were pretreated with either a standard or Gal‐Nac Control‐ASO or Plasminogen‐ASO for 2 weeks prior to tumor implant. Animals were dosed twice a week with Cont‐ASO or Plg‐ASO at either 150 mg·kg^−1^ (standard ASOs) or 7.5 mg·kg^−1^ (Gal‐Nac ASOs) intraperitoneally for the duration of the study as previously described [27, 28]. For subcutaneous studies, Pa03C cells (2.5 × 10^6^) and Pa02C (5 × 10^6^) in 50:50 Matrigel were implanted and monitored by a caliper. Orthotopic studies used Pa03C cells (1.3 × 10^4^) that were implanted into the pancreas following 2 weeks of pretreatment. At necropsy, primary tumors, livers, and lungs were collected and weighed. Tissues were harvested, fixed in 10% neutral buffered formalin, and processed for histological analysis or flash frozen for RNA and protein extraction.

### 2.3 Cell proliferation and soft agar Colony formation assays

Cell growth rates were evaluated as previously described [5]. Briefly, 500 cells of each cell line were seeded in six replicates in a 24‐well plate in complete growth media. At 24, 48, 72, 96 h the plates were washed with 1× PBS before being frozen at −80 °C. All the plates were thawed to room temperature and wells incubated for 5 min with fluorescence dye mixed CyQUANT lysis buffer. The fluorescence intensity was measured at excitation of 480 nm and emission of 520 nm. The doubling time was calculated using the intensities.

A soft agar colony formation assay was performed as defined previously [5]. For the soft agar experiment, 10 000 cells were resuspended in 1 mL 2× complete RPMI, mixed with 1 mL warmed 0.6% agarose and plated over a solidified 1% agarose in 1× RPMI. Following the solidification of the top layer, 2 mL of RPMI was added and cultured at 37 °C for 21 days with continuous medium changes every third day. Colonies were fixed, stained with 0.05% crystal violet, and counted.

### 2.4 Plasma collection and analysis of plasmin generation, plasmin–antiplasmin complexes, fibrinogen, and fibrin degradation products

Whole blood was collected through inferior vena cava of mice into sodium citrate and platelet poor plasma (PPP) was prepared by centrifugation. ELISAs were used to measure fibrinogen (1 : 10 000 diluted PPP; Mouse Fibrinogen ELISA, Immunology Consultants Laboratory Inc., Portland, OR, USA), plasmin‐antiplasmin complexes (1 : 2 diluted PPP; Mouse PAP ELISA, MyBioSource, Inc., San Diego, CA, USA), and fibrin degradation products (undiluted PPP; Asserachrom D‐Di, Diagnostica Stago, Asnières sur Seine, France). Plasmin generation on PPP was performed as previously described [29]. Briefly, trigger solution (TF, phospholipids, and rtPA) was added to reaction wells and α_2_‐macroglobulin‐plasmin to calibrator wells. Reactions were initiated by adding substrate/calcium solution (0.5 mm Boc‐Glu‐Lys‐Lys‐AMC substrate, 16.6 mm CaCl_2_, 60 mg·mL^−1^ bovine serum albumin in 20 mm HEPES, 0.02% NaN_3_, pH 7.3) to diluted plasma. Reactions were monitored every 20 s with a fluorometer (Fluoroskan Ascent, Thrombinoscope, Maastricht, The Netherlands) equipped with a dispenser and 390/460 filter set (excitation/emission). Data were analyzed to yield parameters: lag time, TtPeak, velocity, peak, and endogenous plasmin potential (EPP).

### 2.5 Histology and immunohistochemistry

For the tissue microarray (TMA) study, human PDAC tumor tissue and normal adjacent pancreas sections were obtained from the Indiana University Simon Comprehensive Cancer Center Biospecimen Collection and Banking Core from August 2010 to February 2021. Patients consent was obtained under institutional biobanking collection protocols IUSCC‐0678 Total Cancer Care (IRB: #1807389306), IUCRO‐0280 IUSCC Tissue Bank (IRB: #1106005767) or IUCRO‐0454 (IRB: #1312105608) for use of biological specimens in cancer research. All methods were conducted under standards set by the Declaration of Helsinki. Written informed consent was obtained from each subject and was recorded in a protected database, and methodologies were approved by the IU Human Subjects Office Institutional Review Board. Tumor, pancreas, liver, or lung tissue were fixed n 10% formalin solution overnight and processed into paraffin for sectioning. For immunohistochemistry, tissue slides were stained with primary antibodies for phospho‐H3 (1 : 1000, Millipore Sigma 06570, Burlington, MA, USA), Ki67 (1 : 800, Cell Signaling Technology, cat#12202S), cleaved‐caspase‐3 (1 : 1000, Cell Signaling, Danvers, MA, USA, cat# D175), CD31 (1 : 20, Dianova, cat# SZ31), CD3 (1 : 100, Dianova, Hamburg, Germany, cat# DIA‐303), AIF‐1 (1 : 2000, FujiFilm WAKO Chemicals, Richmond, VA, USA, cat#019‐19 741), Ly6G (1 : 8200, BioXcell, Lebanon, NH, USA, cat# BE0075‐1), fibrinogen (1 : 200, Agilent‐DAKO, Santa Clara, CA, USA, cat# A0080). Positive staining was detected with a biotinylated secondary antibody (Vector Labs Cat#BA‐1000‐1.5 or Vector Labs Cat#BA‐9400‐1.5, Newark, CA, USA), followed by Vectastain ABC (Vector Labs ELITE peroxidase kit – cat# PK‐6100 or Vector Labs Alkaline Phosphatase kit –cat#AK‐5000), and developed using VIP (Vector cat# SK‐4600), SIGMAFAST™ 3,3′‐Diaminobenzidine tablets (Sigma cat# D4293), or SIGMAFAST™ Fast Red TR/Naphthol AS‐MX Tablets (Sigma cat# F4523). Slides were counterstained with Methyl Green (Vector cat# H‐3402‐500) or hematoxylin (Ricca Chemical Company cat# 353032, Arlington, TX, USA). For analysis of KPC tumors, the quantification of the number of positively stained cells or stained areas in 4 non‐overlapping 10× fields were performed using imagej.

For analysis of Pa02C or Pa03C tumors, the number of positively stained cells were determined using Aperio positive pixel count from analysis of whole tissues. To ensure representative areas were captured during each downstream analysis, tumor tissues were sectioned into two halves and each half was either fixed in 10% formalin or flash frozen for protein or RNA analysis. Similarly, the left lobe of each liver was fixed for histology and the remaining tissue was flash frozen. Finally, one of the lungs was fixed in formalin while the other one was flash frozen. For quantitative analysis of metastasis to liver and lungs, the image analysis software halo (Indica Labs, Albuquerque, NM, USA) was used to analyze whole tissue sections to determine the area of metastatic lesions in liver and lungs. This software utilized a machine learning algorithm to identify tumor cells that are present within the tissues of interest. The algorithm detected the tumor cells and calculated those regions as percentage of total tissues using the HALO classifier. A semiquantitative histopathological scoring system was used as an additional approach to quantify spontaneous metastatic lesions in the fixed liver and lung tissues. A score of 0–3 was assigned to H&E‐stained tissue sections based on the number and size of lesions. A score of 0 indicates that the tissue has no metastatic lesions, 0.5 indicates that lesions are very small (10 cells or less) and no more than two lesions are present, 1 indicates that three to five small lesions are present in the tissue, 2 indicates that more than five moderately sized lesions are present within the tissue and finally 3 indicates multiple large lesions present. These were scored by two pathologists with the identity of the sample blinded.

### 2.6 RT‐qPCR

Tumor and liver tissues were homogenized in Trizol Buffer (Invitrogen, Waltham, MA, USA; cat# 15596026) and total RNA was extracted per manufacturer's recommendations. cDNA was synthesized using 1 μg of RNA with High‐Capacity RNA‐to‐cDNA™ Kit (ThermoFisher Scientific, Waltham, MA, USA; cat# 4387406) following the manufacturer's instructions. Quantitative RT‐qPCR was performed with the TaqMan™ Gene Expression Master Mix (ThermoFisher Scientific cat# 4369016) using probes for *Plg* (Mm00447087_m1), *Fga* (Mm00802584_m1), *Egf* (Mm00438696_m1), *Hgf* (Mm01135184_m1), *Fgf2* (Mm01285715_m1), *Tgfb1* (Mm01178820_m1), *Mmp1* (Mm00473485_m1), *Mmp2* (Mm00439498_m1), *Mmp9* (Mm00442991_m1), *Mmp13* (Mm00439491_m1), *Il1b* (Mm00434228_m1), *Il6* (Mm00446190_m1), *Il10* (Mm01288386_m1), *Cdh1* (Mm01247357_m1), *Plgrkt* (Mm00509491_g1), or *S100a10* (Mm00501458_g1). All transcripts level were normalized to the housekeeping internal control *B2m* (Mm00437762_m1). Relative transcript changes were determined using the Pfaffl method.

### 2.7 Western blot for fibrin and EMT marker analysis

Hepatic fibrin(ogen) levels were measured as described [30] after detergent lysis of snap frozen liver and incubation of insoluble protein in reducing conditions (i.e., Urea/DTT/EDTA). Equivalent amounts of reduced insoluble protein, determined using the corresponding soluble protein concentration, were subjected to capillary western blotting (Wes, 12–230 kDa 25‐capillary gels, ProteinSimple, San Jose, CA, USA) for β polypeptide using a chain‐selective antibody (16747‐1‐AP, Proteintech, Chicago, IL, USA) [31]. Quantification of β polypeptide peak area was performed using Compass software (ProteinSimple).

For EMT markers, flash frozen primary tumor tissue was processed and lysed in 1% SDS solution followed by homogenization on ice with a probe sonicator to obtain protein lysates. Protein quantification was performed using the Lowry assay. Equal amounts of proteins (30 μg) were loaded in 4–15% gradient gel (BioRad; cat#4568086) and run in 1X Tris/Glycine/SDS buffer (BioRad, Hercules, CA, USA; cat#1610732) at 80 V for 20 min and 120 V for 60 min. Proteins were transferred to nitrocellulose membranes using the BioRad Trans‐Blot Turbo Transfer System, blocked for 1 h (5% BSA) and incubated overnight with one of the following primary antibodies: E‐Cadherin (1 : 1000, Cell Signaling Technologies #3195), N‐Cadherin (1 : 500, Cell Signaling Technologies #13116), Snail (1 : 1000, Cell Signaling Technologies #3879), Slug (1 : 1000, Cell Signaling Technologies #9585), Vimentin (1 : 1000, Cell Signaling Technologies #3932), Zeb (1 : 1000, Cell Signaling Technologies #70512). After primary antibody incubation, the blots were washed three times for 10 min each using PBS‐Tween and incubated with secondary antibody for 1 h (1 : 10 000 dilution) in 5% BSA. After secondary incubation, blots were washed, and bands imaged using chemiluminescence on the BioRad ChemidocTM MP Imaging System. For increased consistency between tumor samples, bands were quantified against total protein per lane using the biorad image lab software. Background was subtracted and adjusted volume, normalization factor, and normalization volume were generated using the software. Average normalization volume was calculated and plotted to show relative protein expression.

### 2.8 Zymography

KPC cells were seeded in 6 well plates at a density of 1 × 10^6^ cells per well. The following day cells were placed in low‐serum media (i.e., 1% fetal bovine serum) for 24 h followed by stimulation with 1 U·mL^−1^ bovine thrombin (Enzyme Research Lab, South Bend, IN, USA, cat#BT1002a) for 24 h followed by collection of media for analysis. Here, 10 μL of media was mixed with 20 μL of zymography buffer (Bio‐Rad) and 5 mL of this mixture was resolved on a SDS/PAGE gel containing 2 mg·mL^−1^ casein and 20 μg·mL^−1^ human Glu plasminogen (Innovative Research, Novi, MI, USA, cat#IHUPLGGLUAP1MG). As positive controls, 25 ng of purified uPA (Innovative Research, Novi, MI, USA, cat#IMSUPAARCHMW50UG) and tPA (Alteplase, Genentech, San Francisco, CA, USA, cat#NDC 50242‐041‐64) were resolved on the gel. The proteins were renatured through a series of washes in 2.5% Triton X‐100 and enzyme activity developed in 0.1 m Tris–HCl pH 8.1 at 37 °C. The gel was then stained with Coomassie Blue (R‐250), de‐stained, and photographed. The band intensities for uPA and tPA were quantified with imagej.

### 2.9 Statistics

Data were graphed and analyzed in prism graphpad, boston, ma, usa. Tumor volume changes over time were analyzed by repeated measures ANOVA test. Tumor mass, pulmonary foci, RT‐PCR, ELISA, plasmin generation parameters, HALO and EMT marker Western blot data were analyzed by unpaired Mann–Whitney test or *t*‐test, Welch's correction was applied when unequal variances were presented between groups. The statistics for the metastatic lesions using a blinded hand path scoring system quantified the metastatic burden into ordinal levels, therefore we used a one‐sided Mann–Whitney test to compare the tumor burden between pairs of conditions. The *P*‐values were adjusted using fdr.

## 3 Results

### 3.1 KPC PDAC tumors drive plasmin activity and enhance plasmin generation potential

**Fig. 1:**
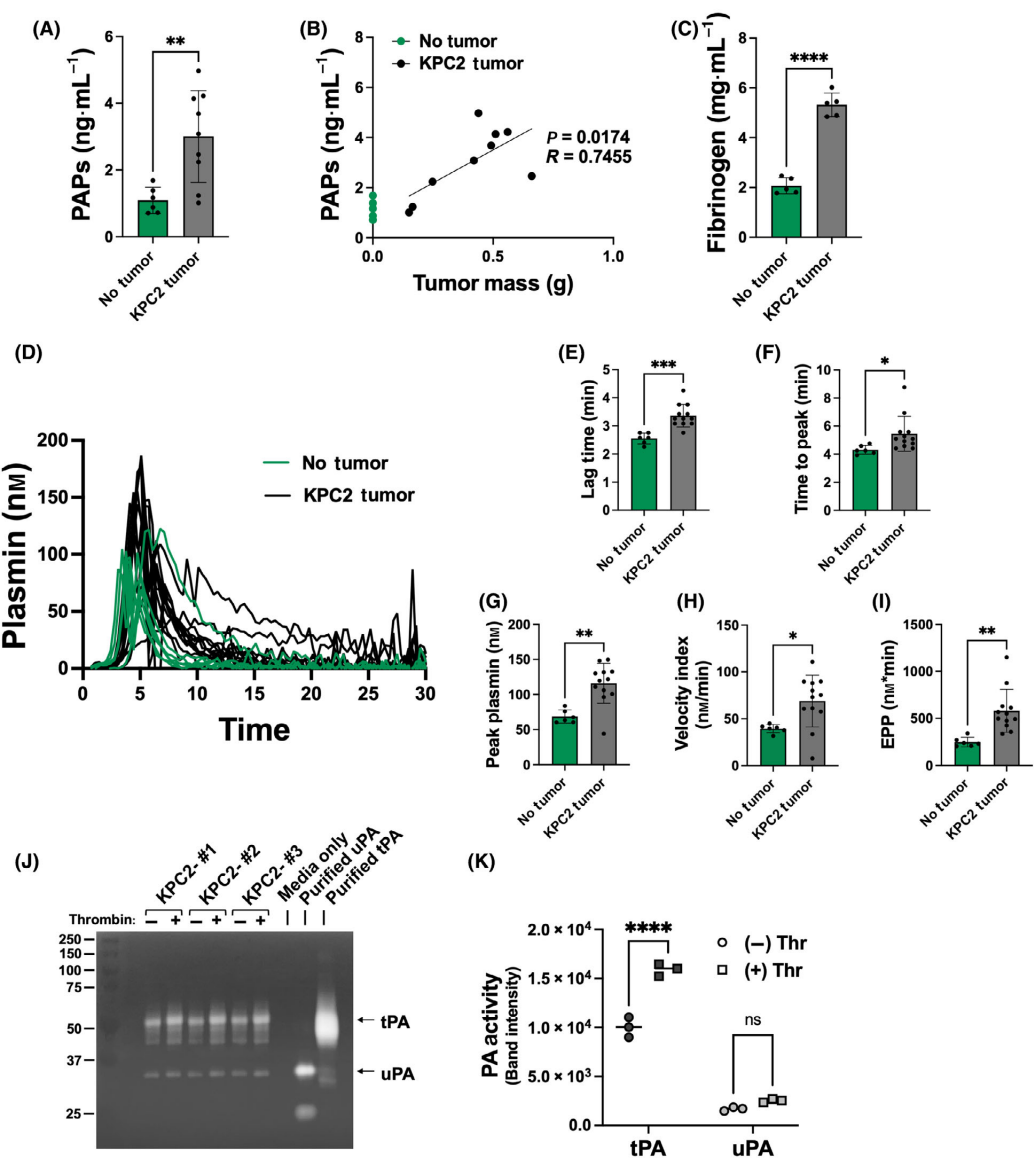
Plasmin activity and plasmin generation potential are each elevated in mice carrying KPC2 PDAC tumors. Plasma was harvested from naïve (no tumor) mice and mice carrying KPC2 tumors that were grown for 3 weeks. (A) Plasma ELISA of plasmin‐antiplasmin complexes (PAPs), *n* = 6 no tumor and *n* = 9 with KPC2 tumor. (B) Correlation analysis of plasma PAP levels versus KPC tumor mass at the time of harvest. (C) Plasma ELISA for fibrinogen, *n* = 5 for each group. Plasmin generation analysis, *n* = 6 no tumor and *n* = 12 with tumor. (D) Individual curves of plasmin generation documenting the concentration of plasmin generated over time. Quantification of the individual parameters of plasmin generation, including (E) lag time, (F) time to peak, (G) peak plasmin, (H) velocity index, and (I) endogenous plasmin potential (EPP). (J) Zymography to analyze urokinase plasminogen activator (uPA) and tissue plasminogen activator (tPA) activity in cell culture media harvested from KPC cells treated with vehicle or 1 U·mL^−1^ thrombin, *n* = 3 per group. Purified tPA (1 μg) and uPA (1 μg) were used a positive controls (K) Quantification of tPA and uPA signals from the zymography gel. Data in bar graphs are expressed as the mean ± standard error of the mean and analyzed by *t*‐test [A, C, E–I, K] or Pearson correlation coefficient analysis [B] for correlation comparisons (*P < 0.05, **P < 0.01, ***P < 0.001, ****P < 0.0001).

To determine whether PDAC tumor growth was associated with an induction in plasmin activity, plasma was harvested from mice bearing orthotopic KPC2 tumors or age‐ and sex‐matched naïve mice. Levels of plasmin‐antiplasmin complexes (PAP), a marker of recent plasmin activity were determined. KPC2 tumor‐bearing mice displayed significantly elevated plasma PAP levels compared to nontumor‐bearing mice (Fig. 1A). Moreover, the concentration of PAPs in plasma significantly and positively correlated with tumor mass (Fig. 1B). In concert with the increase in PAPs, we observed an increase in circulating fibrinogen (Fig. 1C). Fibrinogen is the precursor of fibrin matrix, and fibrin can be a driver of plasmin generation [29]. These observations are consistent with the concept that fibrinogen is an acute phase reactant, and that tumor growth elicits a systemic inflammatory response in the host that may be linked to a tumor driven increase in plasmin activity.

The impact of KPC tumor growth on the host to generate proteolytically active plasmin from plasminogen precursor was also evaluated using a previously described and validated fluorogenic assay [29]. Plotting the individual plasmin generation profiles suggested a slight delay in the initiation of plasmin generation but an overall increase in plasmin activity for plasma samples from KPC tumor bearing mice relative to naïve control animals (Fig. 1D). Indeed, quantification of individual parameters revealed that KPC2 tumor bearing mice had significantly prolonged lag time (Fig. 1E) and time to peak (Fig. 1F) relative to naïve mice. However, the peak plasmin, velocity index, and endogenous plasmin potential (EPP) were each significantly elevated in plasma from KPC2 tumor bearing mice relative to naïve animals (Fig. 1G–I). KPC2 cells were found to be a source of both plasminogen activators. Zymography analysis revealed that KPC2 cells secrete both uPA and tPA and the release of each activator was enhanced by thrombin stimulation (Fig. 1J,K). Collectively, these findings indicate that KPC2 tumors significantly promote plasmin generation and enhance plasmin generating potential in tumor bearing mice possibly through release of tPA and uPA.

### 3.2 Plasminogen deficiency results in reduced PDAC KPC tumor growth and metastatic potential

**Fig. 2:**
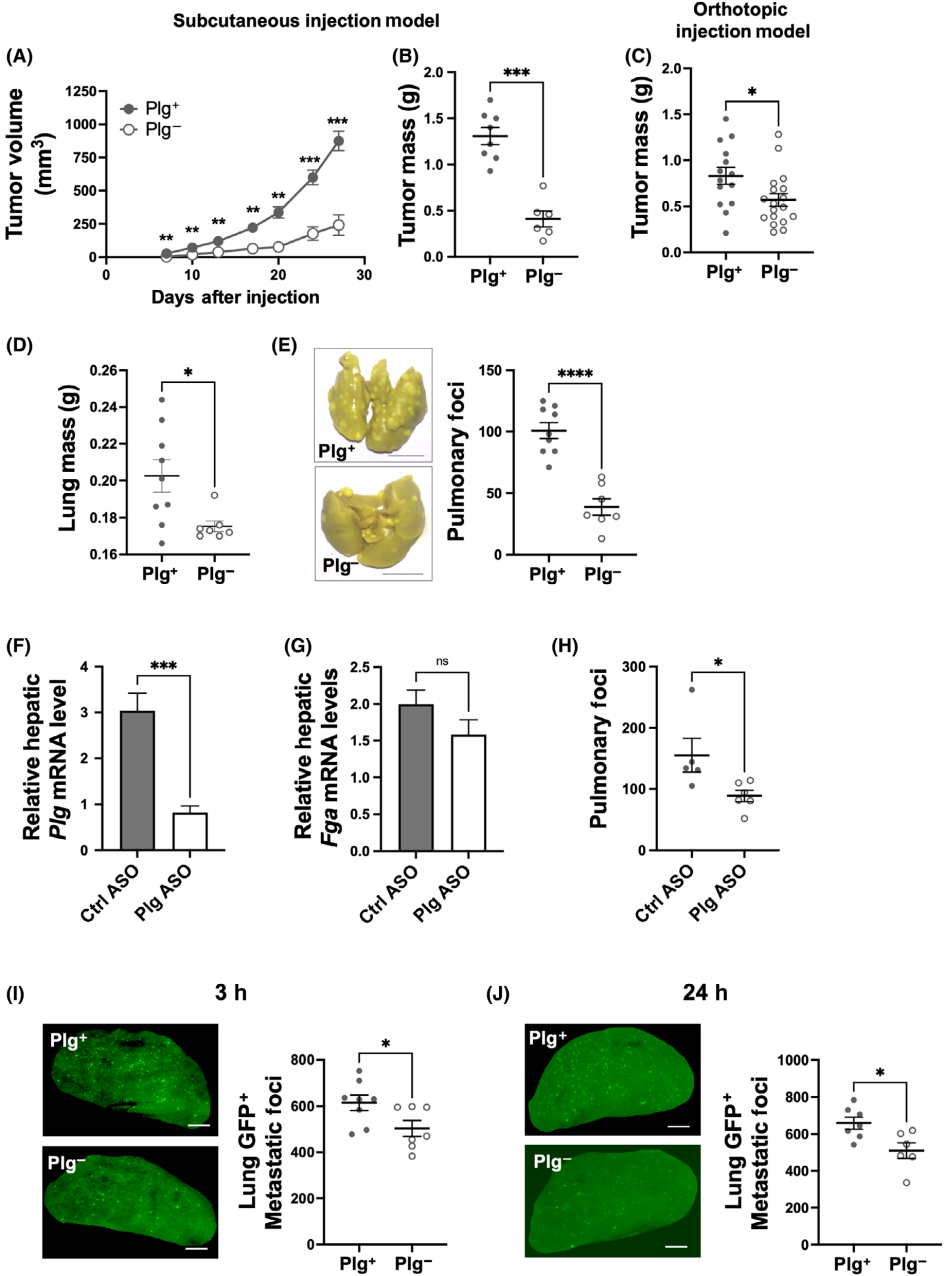
Significantly reduced tumor growth and metastatic potential in plasminogen (Plg)‐deficient mice. Tumor volume (A) and day 28 final tumor mass (B) in Plg^+^ (*n* = 8) and Plg^−^ (*n* = 6) mice following subcutaneous KPC2 injection. (C) Final tumor mass at 3 weeks following orthotopic KPC2 cell injection in Plg^+^ (*n* = 14) and Plg^−^ (*n* = 18) mice. Experimental metastasis evaluating the lungs 3 weeks after tail vein injection of KPC2 PDAC tumor cells into Plg^+^ (*n* = 9) and Plg^−^ (*n* = 7) mice with (D) quantification of lung mass and (E) depiction of representative images of lungs (*left*) and number of surface metastatic nodules (*right*). Scale bar equals 5 mm. Hepatic mRNA level of *Plg* mRNA (F) and *Fga* mRNA (G) as determined by RT‐qPCR in KPC2 tumor bearing mice following treatment with control antisense oligonucleotide (ASO) (*n* = 5) or Plg ASO (*n* = 6). (H) Quantification of the number of surface pulmonary metastatic foci at 3 weeks following tail vein injection with KPC2 cells in mice treated with control ASO (*n* = 5) or Plg ASO (*n* = 6). (I) Representative images (*left*) and quantification of the number of micrometastatic lesions (*right*) in the left lung lobe 3 h following tail vein injection of KPC2 cells in Plg^+^ (*n* = 8) and Plg^−^ mice (*n* = 7). Scale bar equals 1 mm. (J) Representative images (*left*) and quantification of the number of micrometastatic lesions (*right*) in the left lung lobe 24 h following tail vein injection of KPC2 cells in Plg^+^ (*n* = 7) and Plg^−^ (*n* = 6) mice. Scale bar equals 1 mm. Data are presented as mean ± standard error of the mean and were analyzed by Mann–Whitney *U*‐test with *P < 0.05, **P < 0.01, ***P < 0.001, ****P < 0.0001.

To determine if plasmin(ogen) contributes to PDAC primary tumor growth, KPC2 tumor growth studies in Plg^+^ and Plg^−^ mice were performed using both subcutaneous and orthotopic allograft models. KPC2 tumor cells subcutaneously implanted in Plg^+^ mice produced tumors of increasing volume over a 4‐week observation period whereas the same tumor cell suspension injected into Plg^−^ mice yielded tumors of significantly reduced volume (Fig. 2A). This difference translated into a significantly smaller final tumor mass for Plg^−^ mice relative to Plg^+^ mice (Fig. 2B). A similar finding was observed when KPC2 cells were orthotopically injected, with tumors in Plg^−^ mice having a significantly smaller tumor mass compared to orthotopic tumors from Plg^+^ mice (Fig. 2C). The relative reduction in tumor growth secondary to plasminogen deficiency was greater in the subcutaneous model than in the orthotopic model, but the basis of this model‐dependent difference is unknown. A role for plasminogen in KPC2 metastatic potential was next explored using an experimental metastasis assay. Following tail vein injection of KPC2 tumor cells, lung weights were significantly higher for Plg^+^ mice relative to Plg^−^ mice (Fig. 2D). Metastatic KPC2 lesions were readily observed in lung tissue of Plg^+^ mice 21 days after injection and the number of KPC2 metastases was significantly reduced in Plg^−^ mice (Fig. 2E). To verify these findings, circulating plasminogen levels were reduced pharmacologically using an antisense oligonucleotide (ASO) that targets *Plg* mRNA in liver. We confirmed a significant reduction in hepatic *Plg* mRNA levels by 73% (Fig. 2F). Treatment of mice with Plg ASO did not significantly alter hepatic *Fga* mRNA levels, the gene that encodes for the fibrinogen Aα chain (Fig. 2G). Consistent with results of genetic elimination of plasminogen, Plg ASO‐treated mice displayed a significant reduction in metastatic lung lesions compared to control ASO‐treated mice (Fig. 2H), indicating that KPC2 metastatic potential is sensitive to both genetic plasminogen deficiency and pharmacologic plasminogen reduction. To determine if the plasminogen‐dependent differences in the number of metastatic lesions were linked to early events in lung tissue, KPC2‐GFP cells were utilized to evaluate the formation of early micro‐metastatic lesions. At both 3 and 24 h after injection, the number of micro‐pulmonary foci counts were significantly lower in Plg^−^ mice compared to Plg^+^ mice (Fig. 2I,J).

### 3.3 Elimination of plasminogen does not change cell proliferation or EMT but enhances apoptosis in the KPC2 PDAC TME

**Fig. 3:**
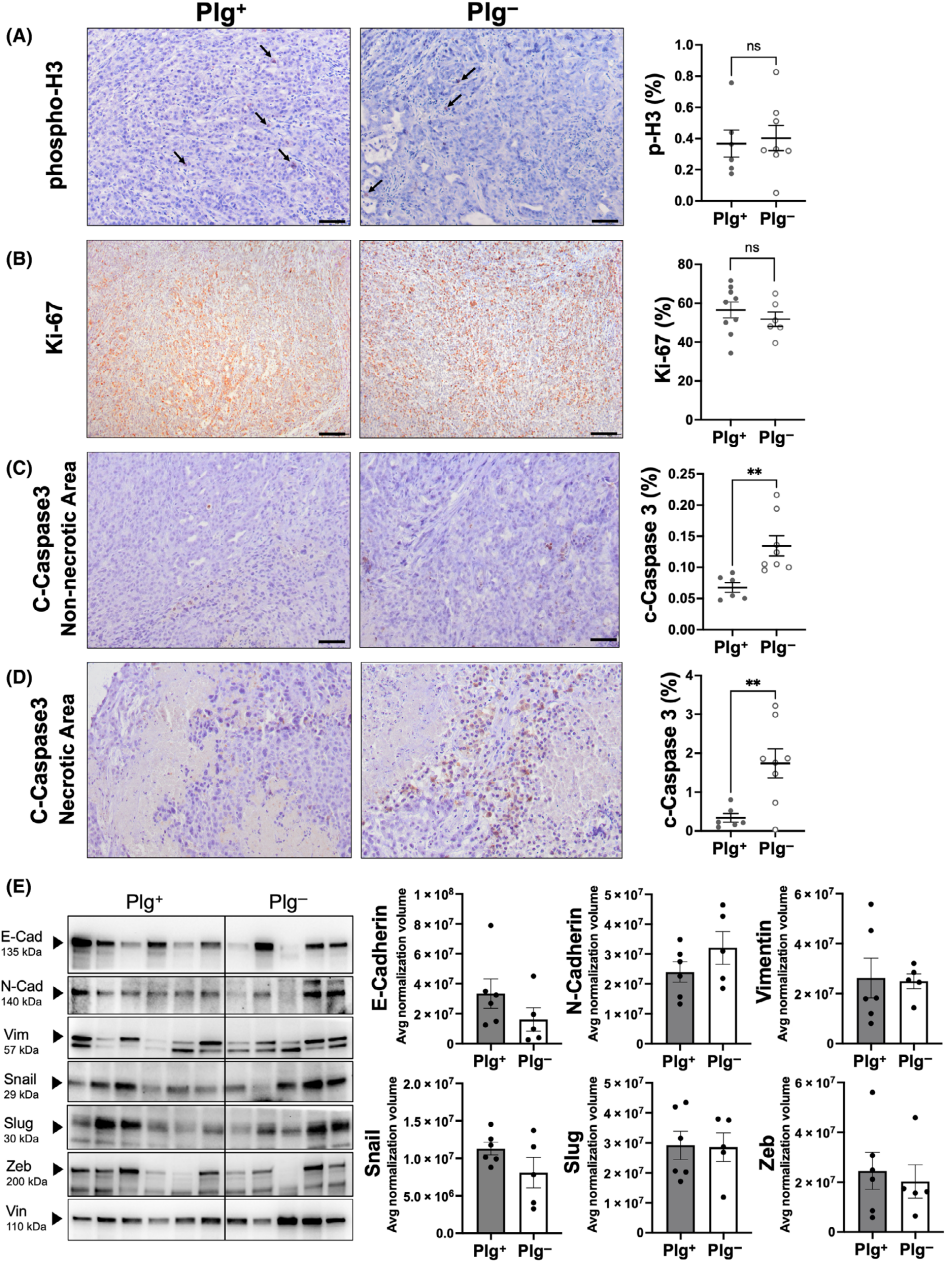
Elimination of plasminogen does not change cell proliferation or epithelial to mesenchymal transition (EMT) but enhances apoptosis in the KPC2 PDAC TME. (A) Representative images (*left*) and quantification (*right*) of phospho‐Histone3 (p‐H3) cell staining in KPC2 PDAC tumor tissue harvested 3 weeks following orthotopic injection into Plg^+^ (*n* = 6) and Plg^−^ (*n* = 8) mice. Arrows indicate positively stained cells. (B) Representative images (*left*) and quantification (right) of Ki‐67 cell staining in tumor tissue harvested 3 weeks following orthotopic injection into Plg^+^ (*n* = 9) and Plg^−^ (*n* = 6) mice. (C) Representative images (*left*) and quantification (*right*) of cleaved caspase‐3 cell staining in non‐necrotic tumor tissue from KPC2 PDAC harvested 3 weeks following orthotopic injection into Plg^+^ (*n* = 6) and Plg^−^ (*n* = 8) mice. (D) Representative images (*left*) and quantification (*right*) of cleaved caspase‐3 cell staining in necrotic tumor tissue harvested 3 weeks following orthotopic injection into Plg^+^ (*n* = 6) and Plg^−^ (*n* = 8) mice. Scale bar = 100 μm. (E) Western blot analyses (*left*) and densitometry quantification (*right*) of epithelial to mesenchymal transition (EMT) markers from tumor tissue 3 weeks following orthotopic injection into Plg^+^ (*n* = 6) and Plg^−^ (*n* = 5) mice. Each lane represents an individual tumor. Densitometry analysis was conducted using total protein normalization. The total protein lysates of each tumor were run three times to generate average normalization volume values. The differences in average normalization volume values for individual tumors from each group were analyzed using a *t*‐test assuming unequal distribution. Data are presented as mean ± standard error of the mean and analyzed by Mann–Whitney test with **P < 0.01.

To begin to dissect the potential mechanisms by which plasminogen promotes tumor growth *in vivo*, histological analyses of KPC2 orthotopic tumors from Plg^+^ and Plg^−^ mice were performed. Analysis of cell proliferation by quantifying the number of phospho‐H3^+^ or Ki‐67^+^ cells revealed no significant differences between KPC2 tumors harvested from Plg^+^ and Plg^−^ mice (Fig. 3A,B). However, analysis of cell apoptosis performed by quantifying the number of cells that were positive for cleaved caspase‐3 indicated that KPC2 tumors from Plg^−^ mice compared to Plg^+^ mice had significantly higher percentages of apoptotic cells both within non‐necrotic areas and within areas of central tumor necrosis (Fig. 3C,D). We next evaluated whether the reduced KPC tumor mass or reduction of metastatic burden observed in Plg^−^ mice was linked to a shift in epithelial‐to‐mesenchymal transition (EMT). The expression of EMT markers showed considerable variation among different samples within the same group. Overall, no significant changes were observed for the EMT markers, N‐Cadherin, Snail, Slug, Zeb, or Vimentin (Fig. 3E). A trend toward reduced E‐Cadherin in tumors from Plg^−^ mice was observed but this did not reach statistical significance. Collectively, these data suggest that cell apoptosis is a primary determinant of reduced KPC2 tumor size in Plg^−^ mice.

### 3.4 Elimination of plasminogen reduces the accumulation of pro‐tumor immune cell populations in the PDAC TME

**Fig. 4:**
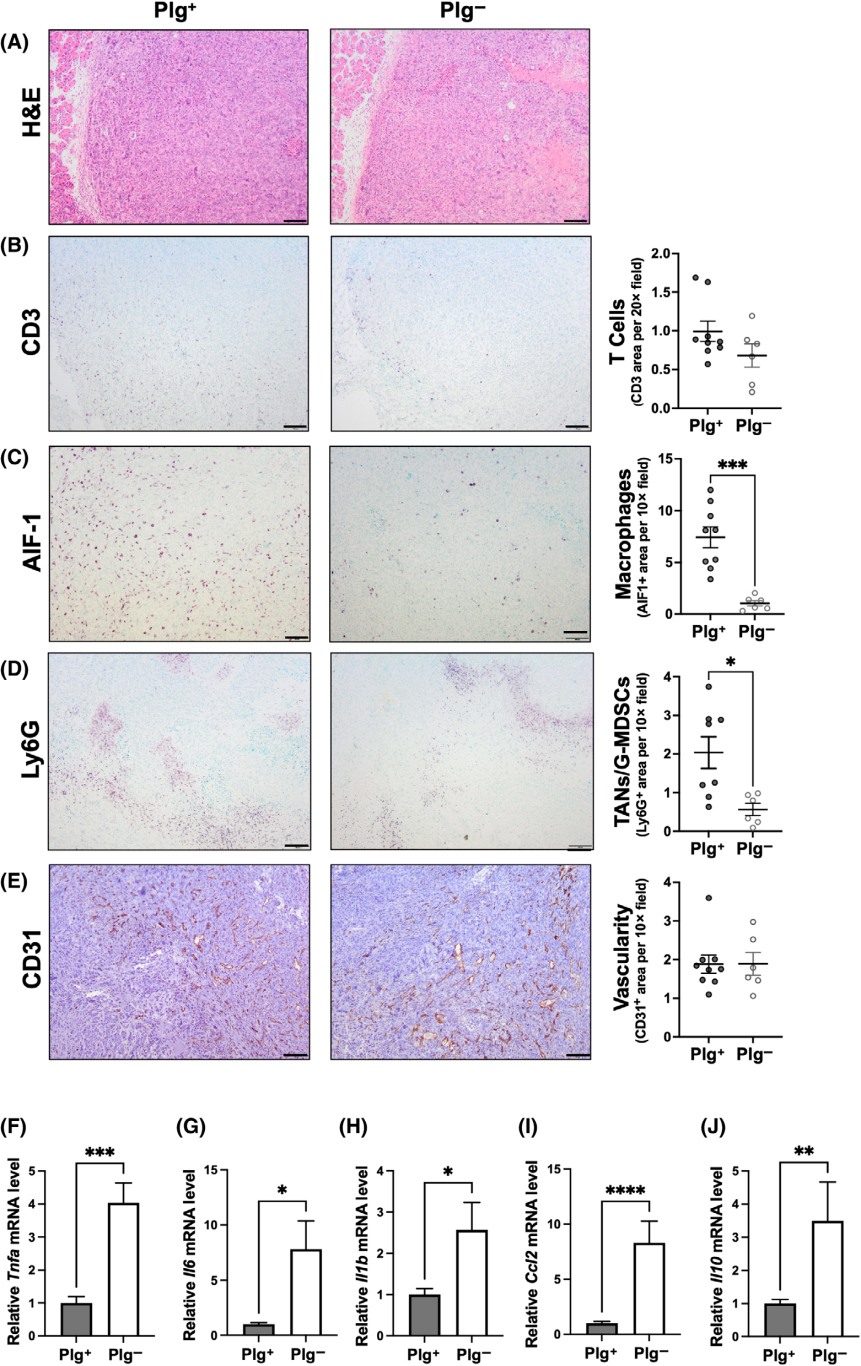
Elimination of plasminogen reduces the accumulation of pro‐tumor immune cell populations in the KPC2 PDAC tumor microenvironment. Representative images (*left*) and quantification (as appropriate) of immunohistochemical staining (*right*) for (A) H&E, (B) CD3, (C) AIF‐1, (D) Ly6G, and (E) CD31 in tumor tissue harvested 3 weeks after orthotopic injection into Plg^+^ (*n* = 9) and Plg^−^ (*n* = 6) mice. Scale bar = 100 μm. Quantitative RT‐PCR analysis of tumor tissue harvested from Plg^+^ (*n* = 14) and Plg^−^ (*n* = 13) mice orthotopically injected with KPC2 cells for (F) *Tnfa*, (G) *Il6*, (H) *Il1b*, (I) *Ccl2*, and (J) *Il10*. Data are presented as mean ± standard error of the mean and were analyzed by Mann–Whitney test with *P < 0.05, **P < 0.01, ***P < 0.001, ****P < 0.0001.

Plasmin(ogen) is best characterized as being the primary fibrinolytic protease responsible for clearing fibrin‐rich blood clots. However, studies have shown that plasmin(ogen) also has roles in mediating leukocyte migration and recruitment of immune cells to the site of injury, through fibrin(ogen)‐dependent and ‐independent mechanisms. In cancer, distinct immune cells have unique roles in anti‐ or pro‐tumor function. To investigate the impact of plasminogen on immune cell populations within pancreatic tumors, analyses of immune cell markers in the KPC2 tumors were performed. Tumor sections stained by H&E revealed no overt differences in tissue organization, morphology, or cellular structure between Plg^+^ and Plg^−^ tumors (Fig. 4A). Total T cell populations as identified by CD3^+^ cells were modestly, but not significantly reduced in Plg^−^ mice compared to Plg^+^ mice (Fig. 4B). More importantly, activated macrophages as identified by allograft inhibitory factor‐1 (AIF‐1) + cells (Fig. 4C) and tumor‐associated neutrophils or granulocytic myeloid‐derived suppressor cells (G‐MDSCs) as identified by Ly6G^+^ cells (Fig. 4D), were found to be significantly decreased in KPC2 tumors from Plg^−^ mice relative to those harvested from Plg^+^ mice, suggesting that plasmin(ogen) promotes recruitment of immune cells that support an immunosuppressive tumor microenvironment. One possible explanation for the observed differences in host cells within the tumors is a genotype‐dependent difference in vascularity. However, no difference in tumor vasculature was observed based on quantification of CD31^+^ area within tumors (Fig. 4E), suggesting plasminogen‐deficiency did not alter tumor angiogenesis.

The local production of inflammatory mediators was also analyzed in KPC2 tumors isolated from Plg^+^ and Plg^−^ mice. Here, the mRNA levels of proinflammatory cytokines *Tnfa*, *Il6*, *Il1b* as well as the chemokine *Ccl2* (Fig. 4F–J) were significantly higher in Plg^−^ tumors relative to Plg^+^ tumors consistent with the smaller tumor size and increased levels of apoptosis. Intriguingly, local mRNA levels of the anti‐inflammatory cytokine *Il10* were also increased in Plg^−^ compared to Plg^+^ tumors, although the fold difference appeared to be generally less than that observed for the proinflammatory markers.

### 3.5 Fibrin is present in the PDAC TME, but fibrinogen deficiency does not alter orthotopic KPC2 PDAC tumor growth

**Fig. 5:**
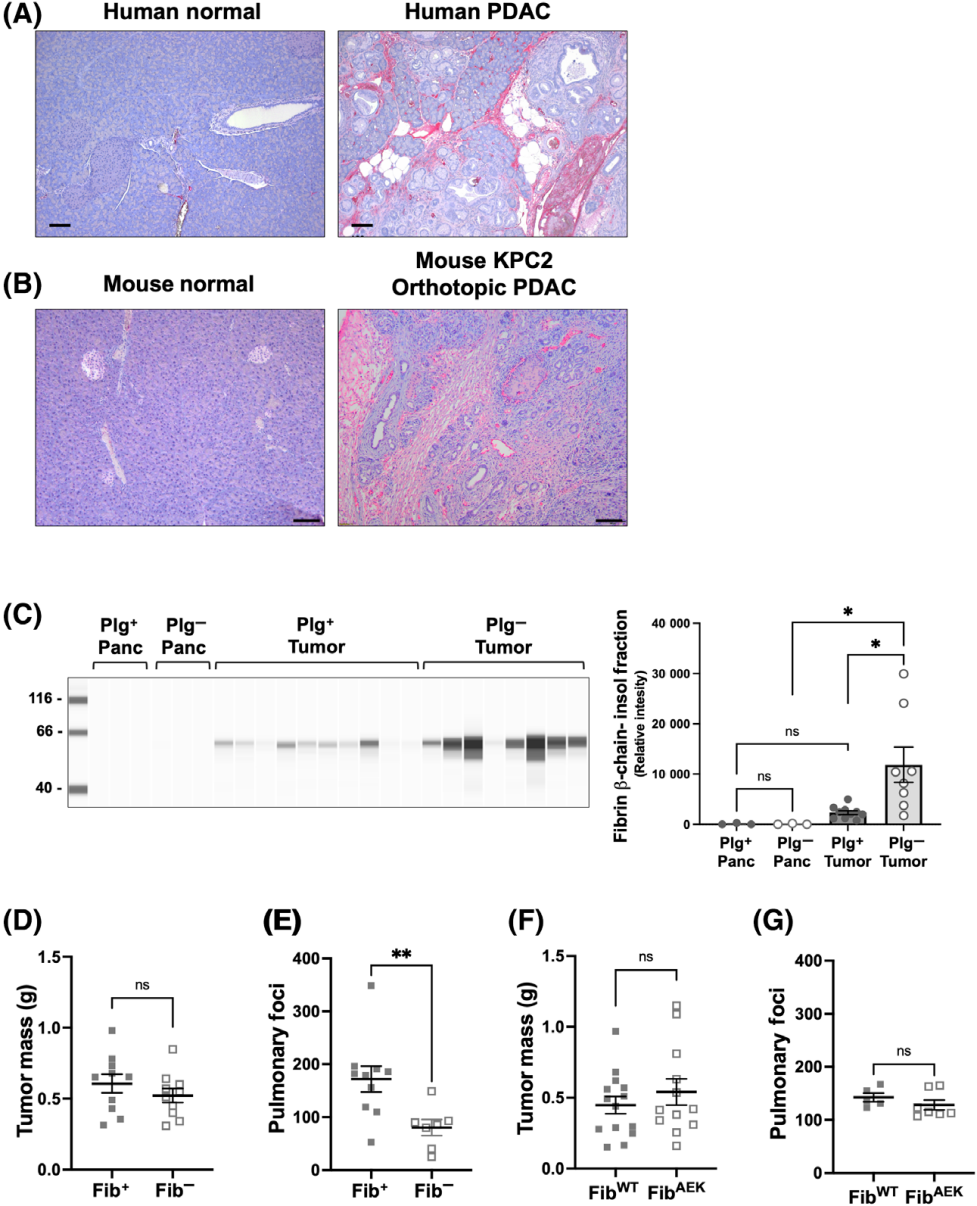
Extravascular fibrin(ogen) deposition is a consistent feature of the PDAC tumor microenvironment, but genetically eliminating fibrinogen or fibrin polymer formation does not change KPC2 PDAC tumor growth. Immunohistochemical staining for fibrin(ogen) in tissue sections of (A) human normal human pancreas and PDAC patient biopsies and (B) normal wildtype mouse pancreas and orthotopic KPC2 cell PDAC tumor tissue from a WT mouse. Scale bar = 100 μm. (C) Quantification of the fibrin content in normal pancreas (Pan) (*n* = 3 per group) or tumor tissue (*n* = 10 for Plg^+^ and *n* = 8 for Plg^−^) at 3 weeks following orthotopic injection of KPC2 cells in Plg^+^ and Plg^−^ mice. Data are presented as mean ± standard error of the mean and analyzed by 1‐way ANOVA with Dunn's multiple comparisons test (*P < 0.05). (D) Tumor mass 3 weeks after orthotopic injection of KPC2 cells into Fib^+^ (*n* = 10) and Fib^−^ (*n* = 10) mice. (E) Number of surface pulmonary foci in Fib^+^ (*n* = 10) and Fib^−^ (*n* = 7) mice 3 weeks following tail vein injection of KPC2 cells. (F) Tumor mass 3 weeks after orthotopic injection of KPC2 cells into Fib^WT^ (*n* = 14) and Fib^AEK^ (*n* = 12) mice. (G) Number of surface pulmonary foci in Fib^WT^ (*n* = 5) and Fib^AEK^ (*n* = 7) mice 3 weeks following tail vein injection of KPC2 cells. Data are presented as mean ± standard error of the mean and were analyzed by Mann–Whitney test with **P < 0.01.

Given that fibrin can serve as a critical co‐factor for plasmin generation and is a primary proteolytic target of plasmin, we next evaluated whether extravascular fibrin(ogen) is a stromal component of the PDAC tumor microenvironment. Previous studies have identified fibrin deposition in the PDAC TME [32]. Here, of the 25 PDAC tissues analyzed in a human tumor microarray (TMA), 24 of the PDAC tissues stained positively for fibrin (Fig. S1). In separate analyses of patient biopsies, fibrin was absent from normal pancreas but was readily detected in PDAC tissue (Fig. 5A). Similarly, normal pancreas tissue from WT mice had no fibrin staining whereas tumor tissue following orthotopic KPC2 tumor cell injection and growth had robust staining (Fig. 5B). These findings suggest that local coagulation system activation occurs in human PDAC and is recapitulated in the orthotopic KPC mouse model. The relative accumulation of fibrin in the orthotopic KPC2 microenvironment was directly quantified from tumors grown in Plg^+^ and Plg^−^ mice. Whereas normal pancreas tissue was devoid of fibrin in mice of each genotype, fibrin was readily detected in KPC2 tumors with significantly higher levels of fibrin in Plg^−^ mice compared to Plg^+^ mice (Fig. 5C).

The presence of fibrin in the PDAC TME suggested the hypothesis that plasmin may be promoting PDAC tumor growth and metastatic potential by clearing fibrin matrices. An extension of this hypothesis is that eliminating fibrin(ogen) itself would enhance PDAC tumor growth and metastasis. To determine whether fibrin(ogen) contributes to PDAC tumor growth or metastatic potential, orthotopic tumor growth allograft and experimental metastasis assays were performed in mice carrying mutations in the fibrinogen gene locus. Here, fibrinogen‐deficient mice (Fib^−^) or Fib^+^ control mice were implanted with KPC2 cells, but no difference in primary KPC2 tumor growth was observed in the orthotopic model (Fig. 5D). A statistically significant diminution in KPC2 metastatic lung foci was observed in Fib^−^ mice compared to Fib^+^ mice (Fig. 5E). This finding is consistent with previous studies documenting a role for fibrin(ogen) in experimental metastasis assays for other C57Bl/6‐derived tumor cell types (e.g., Lewis lung carcinoma and B16 melanoma) [33, 34]. We next evaluated whether tumor growth and metastasis were affected in Fib^AEK^ mice expressing normal levels of fibrinogen that is unable to be converted to fibrin matrix. Both KPC2 primary tumor growth and experimental metastasis were equally robust in control Fib^WT^ and Fib^AEK^ mice with no statistically significant differences in tumor mass nor in the number of lung metastatic lesions (Fig. 5F,G). These data suggest that the conversion of fibrinogen to fibrin matrix is not required to promote KPC2 metastatic potential.

### 3.6 Reduction of plasminogen receptor‐KT or the plasminogen receptor S100A10 from KPC2 tumor cells reduces primary tumor growth but differentially impact metastatic potential

**Fig. 6:**
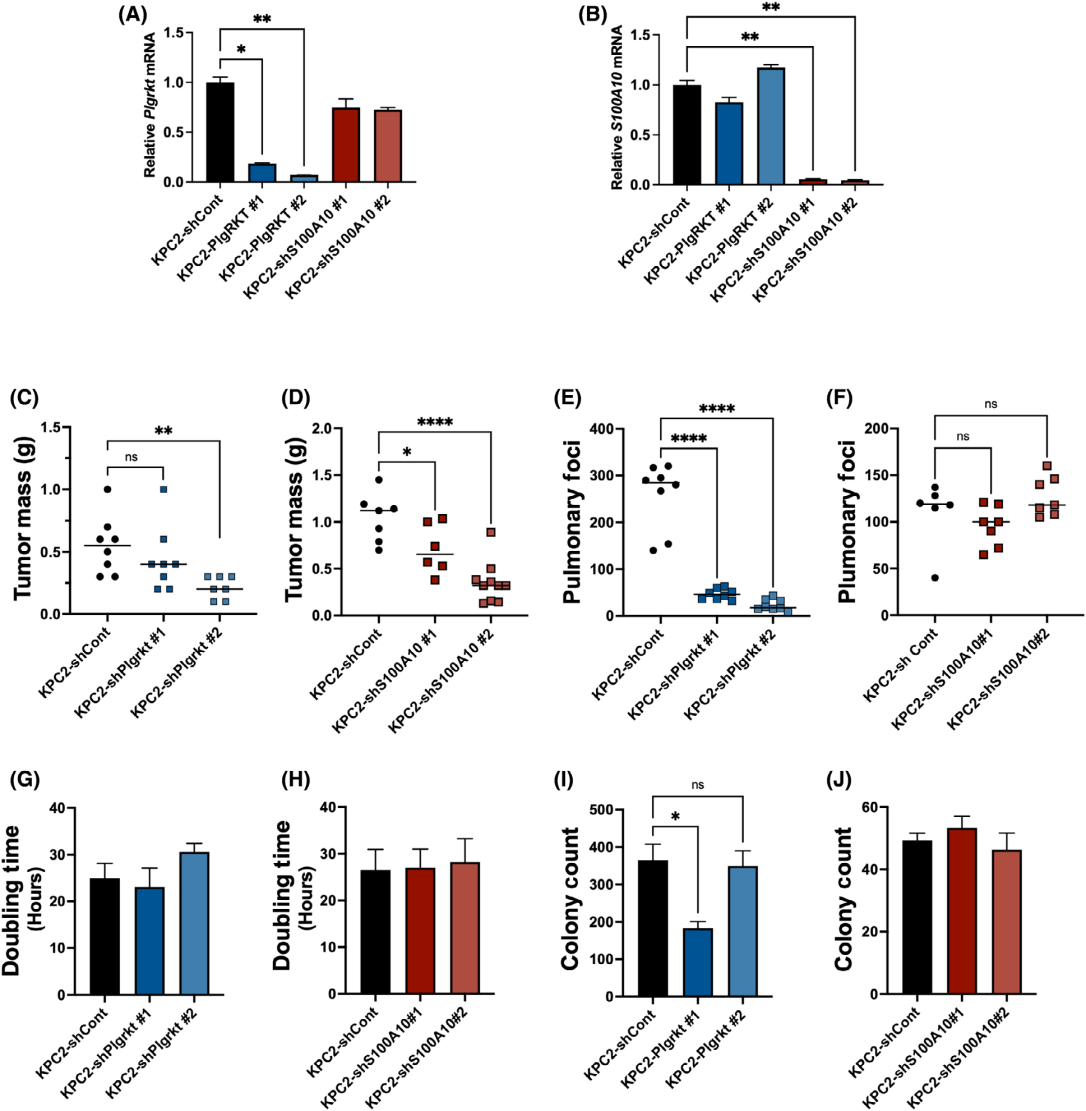
Reduction of the plasminogen receptors Plg‐RKT or S100A10 in KPC2 tumor cells differentially impacts tumor growth and metastatic potential. RT‐qPCR analysis of (A) *Plgrkt or* (B) *S100a10* of mRNA harvested from KPC2 cells transduced with a control lentivirus (KPC2‐shCont), lentivirus expressing a shRNAs targeting *Plgrkt*, or lentivirus expressing a shRNAs targeting *S100a10* (*n* = 3 replicates for each group). (C) Tumor mass after orthotopic injection into WT mice with KPC2‐shControl (*n* = 8) cells or KPC2‐shPlgrkt (*n* = 8 and 7, respectively) pools. (D) Tumor mass after orthotopic injection into WT mice with KPC2‐shControl (*n* = 7) cells or KPC2‐shS100A10 (*n* = 6 and 9, respectively) pools. (E) Number of surface pulmonary foci in WT mice following injection with KPC2‐shControl (*n* = 8) cells or KPC2‐shPlgrkt (*n* = 8 and 8, respectively) pools following tail vein injection of KPC2 cells. (F) Number of surface pulmonary foci in WT mice following injection with KPC2‐shControl (*n* = 6) cells or KPC2‐shS100A10 (*n* = 7 and 7, respectively) clones following tail vein injection of KPC2 cells. (G) *In vitro* cell doubling time analysis of KPC2‐shControl cells and KPC2‐shPlgrkt pools (*n* = 3 replicates per group). (H) *In vitro* cell doubling time analysis of KPC2‐shControl cells and KPC2‐shS100A10 pools (*n* = 3 replicates per group). (I) Soft agar colony formation of KPC2‐shControl and KPC2‐shPlgrkt pools (*n* = 3 replicates per group). (J) Soft agar colony formation of KPC2‐shControl and KPC2‐shS100A10 pools (*n* = 3 replicates per group). Data are presented as mean ± standard error of the mean and were analyzed by one‐way ANOVA with a Tukey's Multiple Comparison Test with *P < 0.05, **P < 0.01, ****P < 0.0001.

We next evaluated whether tumor growth and metastasis were linked to the expression of plasminogen receptors by KPC2 cells. Here, we analyzed both plasminogen receptor‐KT (Plg‐RKT) and S100A10. Plg‐RKT plays a critical role in binding and activation of plasminogen via its C‐terminus lysine residues on cell surfaces [35] whereas S100A10 is a component of the S100A10/Annexin A2 plasminogen receptor complex [36]. KPC2 pools were generated in which Plg‐RKT or S100A10 were knocked down using shRNA. Two distinct pools of cells for each target were identified. Quantitative RT‐PCR analysis confirmed that *Plgrkt* mRNA was significantly reduced by ~80% and 95%, respectively (Fig. 6A), and *S100A10* mRNA was reduced by 92% and 94%, respectively (Fig. 6B). Notably, shRNA‐mediated reduction of one receptor did not alter mRNA levels of the other (Fig. 6A,B). When Plg‐RKT expression was decreased by 80% in the tumor cells (KPC2‐shPlgrt#1), there was a trend toward smaller KPC2 orthotopic tumors but with 95% reduction (KPC2‐shPlgrkt#2) significantly smaller tumors were observed (Fig. 6C). Similarly, significantly smaller tumors were observed with reduction of *S100a10* in both pools generated (Fig. 6D). KPC2 metastatic potential was particularly sensitive to loss of *Plgrkt* as both pools showed a striking and significant diminution in the number of lung metastatic tumor nodules (Fig. 6E). In contrast, reduction of S100A10 in KPC2 cells had no impact on their metastatic potential to the lungs (Fig. 6F). No change in the *in vitro* doubling time (Fig. 6G,H) was observed with reduction of *Plgrkt* or *S100a10*. A modest reduction in soft agar colony formation was observed for one of the pools in which *Plgrt* was reduced but not for either of the *S100a10* pools (Fig. 6I,J). Given the trend in reduction E‐cadherin expression in KPC2 tumors grown in Plg^−^ mice (Fig. 3E), we analyzed expression of the gene for E‐cadherin (*Cdh1*) in KPC2 cells grown *in vitro* and found that sufficient reduction of either receptor resulted in a modest but statistically significant reduction in *Cdh1* mRNA (Fig. S2C,D). Finally, we also examined the potential contribution of host‐derived Plg‐R_KT_. Orthotopic tumor growth of KPC2 and metastatic potential were not different in either control (Plg‐RKT^+/+^) or Plg‐RKT‐deficient (Plg‐RKT^−/−^) mice (Fig. S3A,B, respectively).

### 3.7 Plasminogen reduction significantly reduces tumor mass of human low‐passage PDAC tumor cell lines

**Fig. 7:**
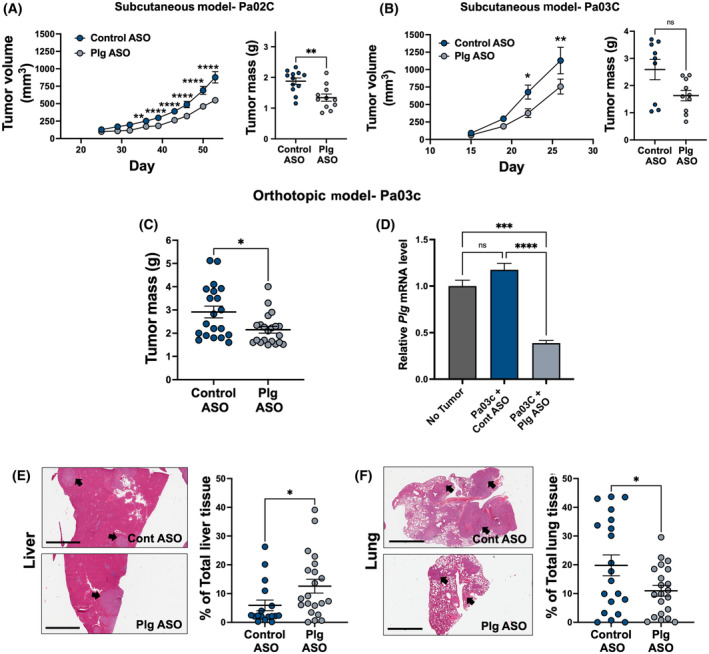
Partial depletion of circulating plasminogen reduces primary tumor growth and impedes metastasis in human PDAC xenograft models. (A) Tumor volume (*left*) and mass (*right*) of human Pa02C subcutaneous tumors in immunodeficient NSG mice treated with Control (*n* = 12) or Plg (*n* = 11) antisense oligonucleotide (ASO). (B) Tumor volume (*left*) and mass (*right*) of human Pa03C subcutaneous tumors in NSG mice treated with Control (*n* = 9) or Plg (*n* = 10) ASO. (C) Tumor mass 5 weeks after orthotopic injection of Pa03C cells into mice treated with Control ASO (*n* = 20) or Plg (*n* = 21) ASO. (D) Quantitative RT‐PCR analysis for *Plg* expression in liver harvested from naïve NSG mice (*n* = 3) or NSG mice orthotopically injected with Pa03C cells and treated with control ASO (*n* = 11) or Plg‐ASO (*n* = 9). (E) Representative H&E‐stained histological sections (*left*) and histology quantitation (*right*) of Pa03C metastases within liver tissue from the same mice analyzed in (C). (F) Representative H&E‐stained histological sections (*left*) and histology quantitation (*right*) of Pa03C metastases within lung tissue from the same mice analyzed in (C). Arrows indicate metastatic lesions. Scale bar = 2 mm. Histological analyses of metastatic lesions are represented as the percent of total tissue sectioned. Data are presented as mean ± standard error of the mean and analyzed using *t*‐test assuming unequal distribution [B, C, E, F] or by one‐way ANOVA with a Tukey's Multiple Comparison Test [A, D] with *P < 0.05, **P < 0.01, ***P < 0.001, ****P < 0.0001.

To determine whether the growth and metastasis of patient‐derived PDAC tumor cells was sensitive to plasminogen reduction, we performed xenograft studies using previously described low‐passage patient‐derived human PDAC cell lines [37, 38]. Reduction of circulating plasminogen using a specific Plg ASO significantly reduced subcutaneous tumor volume over time (Fig. 7A) and the final tumor mass (Fig. 7A) of human Pa02C tumors relative to control ASO treated mice. Using an additional low‐passage patient‐derived PDAC cell line Pa03C, significantly reduced tumor volume was observed on treatment with Plg‐ASO (Fig. 7B). Although reduction in tumor mass did not reach statistical significance in this model, there was a trend toward reduction (Fig. 7D, *P* = 0.081).

Studies were extended to evaluate orthotopic Pa03C tumor growth. Here, the tumor mass at the time of harvest was significantly less in Plg ASO treated mice compared to control ASO‐treated animals (Fig. 7C). Analyses of liver tissue at the time of tumor harvest indicated that *Plg* mRNA levels in Plg ASO treated mice were reduced by 67% relative to Control ASO treated animals (Fig. 7D). Plasma isolated from mice at the time of tumor harvest had similar levels of PAPs across all tumor‐bearing groups and these levels were significantly elevated relative to plasma from mice without tumors (Fig. S4A). However, both naïve and tumor‐bearing mice had similar circulating fibrinogen levels (Fig. S4B). Plasmin generation analysis revealed that plasma from Plg ASO treated animals was devoid of plasmin‐generating potential (Fig. S4C). Plasma from mice receiving no ASO or Control ASO displayed plasmin generation profiles similar to animals without tumors (Fig. S4C). Spontaneous metastasis was also observed in mice with orthotopic Pa03C tumor growth. Histological assessment indicated that liver metastatic lesions were higher on Plg ASO treatment, which was analyzed using quantitative image analysis by HALO (Fig. 7E) and semiquantitative pathology scoring of individual liver tissues (Fig. S5A). However, the metastatic lesions to the lungs were significantly lower on treatment with Plg‐ASO (Fig. 7F, Fig. S5B).

**Fig. 8:**
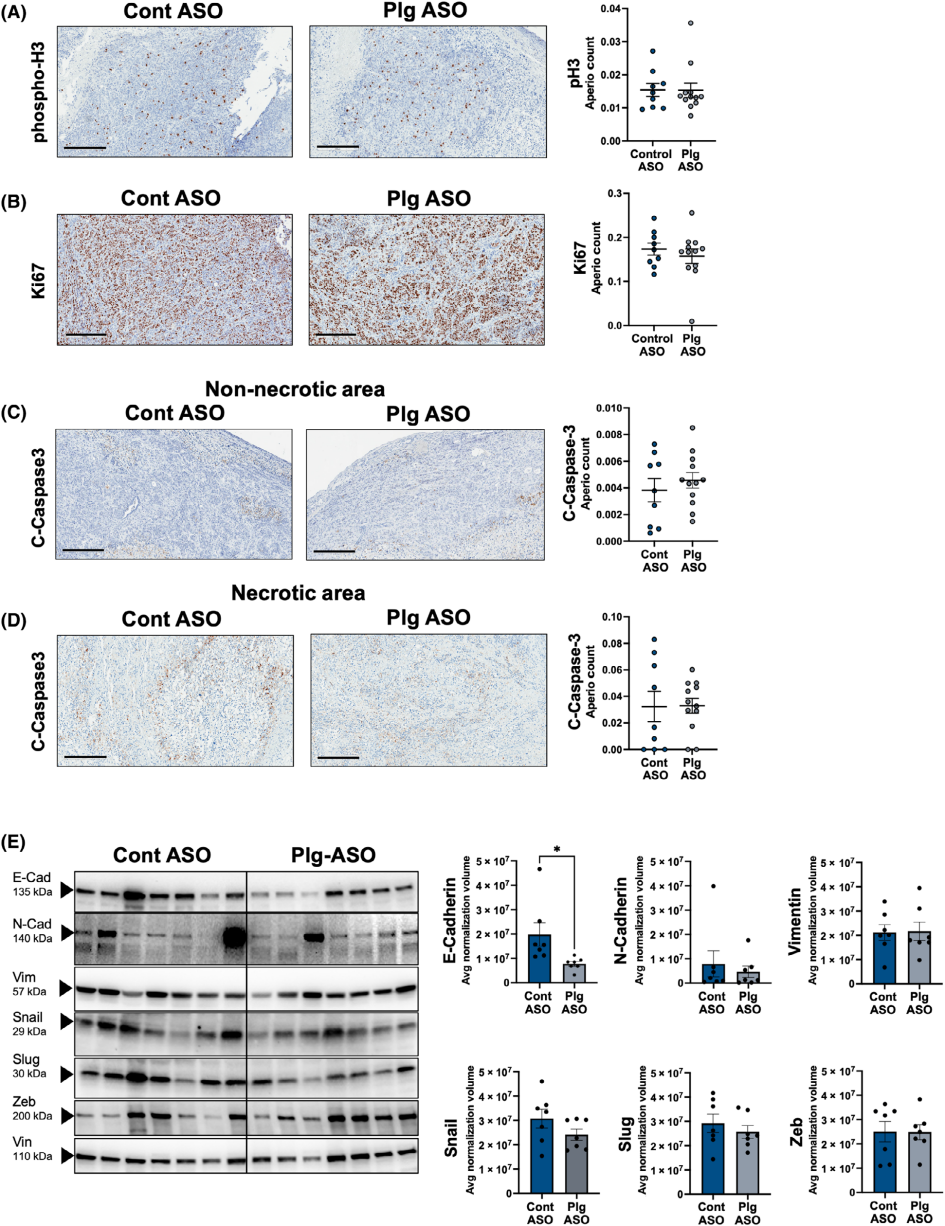
Partial depletion of plasminogen did not impact proliferation or apoptosis markers, but reduced epithelial to mesenchymal (EMT) marker, E‐cadherin in orthotopic human PDAC tumors. Representative images (*left*) and quantification (*right*) of Pa03C orthotopic tumor tissue harvested from control (*n* = 9) and Plg (*n* = 12) antisense oligonucleotide (ASO) treated mice for proliferation markers (A) phospho‐H3 and (B) Ki67 as well as the apoptosis marker cleaved caspase‐3 in (C) non‐necrotic tumor and (D) necrotic regions. Scale Bar = 300 μm. (E) Western blot analyses (*left*) and densitometry quantification (*right*) of EMT markers from Pa03C tumor tissue 5 weeks following orthotopic injection into NSG mice treated with control (*n* = 7) or Plg (*n* = 7) ASO (*P < 0.05). Each lane represents an individual tumor. Densitometry analysis was conducted using total protein normalization. The total protein lysates of each tumor were run three times to generate average normalization volume values. Data are presented as mean ± standard error of the mean and analyzed using *t*‐test assuming unequal distribution with *P < 0.05.

Quantitative histological analyses of the tumor tissues were also performed. No differences in cell proliferation in tumor tissue were observed based on phospho‐H3 (Fig. 8A) and Ki67 staining (Fig. 8B). Cleaved caspase‐3 analysis of non‐necrotic (Fig. 8C) and necrotic (Fig. 8D) tumor revealed no differences suggesting that reduced tumor growth in Plg ASO treated mice was not due to increased apoptosis by caspase‐3 cleavage. Finally, analysis of EMT markers revealed that EMT marker E‐cadherin was significantly reduced by 2.6‐fold in tumors from mice treated with Plg ASO relative to control ASO treated mice (Fig. 8E). Although the reduction in E‐cadherin expression is statistically significant, a corresponding increase in N‐cadherin was not observed suggesting that EMT is not driving the tumor phenotype in either group at the time of sacrifice. No differences in Snail, Slug, Zeb, or Vimentin were observed (Fig. 8E). Collectively, these findings are similar to those observed in the KPC tumor model and suggest that plasminogen activation in the TME enhances both tumor growth and metastasis, especially metastatic burden in the lung.

## 4 Discussion

Biomarkers of plasmin‐mediated proteolysis suggest plasmin is active in patients with PDAC. Specifically, patient studies focusing on the plasmin‐generated fibrin degradation product D‐dimer have indicated that elevated D‐dimer levels are associated with a worse overall survival for several solid tumor malignancies, including PDAC [39, 40]. In one recent study of 1351 patients undergoing radical surgical resection of PDAC, high preoperative D‐dimer levels were strongly associated with a 6.3‐month reduction in overall survival (i.e., 15.0 vs 21.3 months) [41]. Intriguingly the relationship between PDAC disease severity and D‐dimer is not totally clear cut. In a second study, it was found that for 62 patients undergoing surgery for confirmed PDAC without detectable venous thrombosis, high levels of D‐dimer in the portal vein were associated with significantly longer overall survival [42]. Our studies revealed robust plasmin generation in mice with either syngeneic KPC tumors or patient‐derived xenograft tumors as evidenced by high levels of PAPs. Plasma PAP levels were positively correlated with KPC tumor size, supporting the concept that plasmin promotes tumor growth. Moreover, plasmin generation potential was significantly elevated in mice bearing KPC tumors suggesting these PDAC tumors established a very potent plasminogen activation state. In preliminary analyses, we did not observe increased fibrin degradation products (FDPs) or a correlation between FDPs and tumor mass (data not shown), collectively suggesting that although PDAC drives plasmin generation, fibrinolysis may not be the mechanism.

Several studies suggest that the plasminogen activators uPA [21, 43] and tPA [20, 44, 45] are linked to PDAC pathogenesis. Consistent with these reports, we show that KPC PDAC tumor cell secrete both uPA and tPA. However, no studies have directly evaluated the contribution of plasminogen itself to PDAC and analyses of the potential contribution of plasmin(ogen) to tumor growth of other cancer types is limited. Current reports have generally shown that primary tumor growth is independent of plasminogen. For example, the growth of spontaneous breast tumors in polyoma virus middle T antigen (PyMT)‐expressing mice was unaffected by plasminogen deficiency [23]. In addition, a study examining the direct injection of B16F10 melanoma cells into the brain striatum revealed identical tumor growth between Plg^+^ and Plg^−^ mice [46]. Tumor growth of both Lewis lung carcinoma (LLC) and T241 fibrosarcoma cells was unaffected by plasminogen deficiency following injection into the dorsal skin of mice but was significantly reduced in plasminogen‐deficient mice if tumor cells were injected into the footpad [47]. These data suggest anatomical location played a role in the plasminogen‐dependent tumor growth. In addition, the growth of chemically induced skin tumors was reduced in Plg^−^ mice [48]. In these later two scenarios, growth reduction was linked to exuberant peritumoral fibrin formation restricting growth [23, 42]. Thus, a significant finding from these studies is that plasminogen deficiency or reduction significantly diminished KPC PDAC tumor growth but fibrinogen deficiency did not.

The reduction in KPC PDAC primary tumor growth in Plg^−^ mice was linked to increased apoptosis (i.e., cleaved caspase‐3^+^ cells) throughout the tumor. Reduced tumor size was also associated with elevated expression of proinflammatory cytokines and reduced infiltration of tumor‐supporting immune cells, including macrophages and G‐MDSCs in Plg^−^ tumors. In contrast, local markers of EMT were not changed on plasminogen depletion. Previous *in vitro* and *in vivo* studies have shown that plasmin(ogen) supports macrophage migration in several contexts [49]. Moreover, the ability of plasmin(ogen) to support macrophage migration, including tumor‐supporting macrophages, has been linked to the expression of plasminogen receptors. For example, mice deficient in the plasminogen receptor S100A10 did not support tumor growth of subcutaneously injected LLC and fibrosarcomas [50]. Reduced tumor growth was linked to reduced macrophage infiltration to the tumors secondary to loss of S100A10 expression by the macrophages [50]. Plg‐RKT has been shown to support macrophage migration [24, 51]. However, our studies showed that PDAC tumor growth was unaffected by Plg‐RKT deficiency in the host. This may be due to Plg‐RKT being expressed only on a subset of macrophages, namely proinflammatory macrophages [51]. Thus, in the context of PDAC, Plg‐RKT may not be expressed by or contribute to the accumulation of tumor‐supporting macrophages within PDAC tumors.

A significant reduction in KPC primary tumor growth and metastatic potential following reduction of *Plgrkt* expression and *S100a10* in KPC tumor cells using shRNA was observed. The relative reduction in both primary growth and metastasis with *Plgrkt* reduction was roughly equivalent to that observed with host plasminogen deficiency. The reduction in KPC tumor growth with suppression of *Plgrkt* or *S100a10* expression in the tumor cell is consistent with low tumor expression of these genes in human PDAC being associated with prolonged patient survival (see Fig. S2A,B). Others have reported that high expression of the plasminogen receptors S100A10 or α‐enolase‐1 in PDAC are associated with poor prognosis [52, 53]. Pharmacological inhibition of α‐enolase‐1 using an inhibitory antibody suppressed plasminogen‐dependent invasion of human PDAC cells, and their metastatic spreading in mice [52]. Moreover reduction of S100A10 in PANC‐1 reduced orthotopic tumor growth in mice [54]. Collectively, these findings indicate that linking plasmin(ogen) to the tumor cell surface through multiple receptors can promote PDAC tumor growth.

Elimination of plasminogen significantly reduced the metastatic potential of KPC PDAC tumor cells and reduced metastasis of orthotopic Pa03C tumors. Indeed, a reduced number of KPC metastatic lesions in lung tissue was observed in Plg^−^ mice relative to Plg^+^ mice following tail vein injection. The reduction in the number of micro‐metastatic lesions in as short as 3 h after injection in Plg^−^ mice suggested that the loss of plasminogen conferred a reduction in the initial adhesion and/or survival of KPC tumor cells within the lung. These findings are consistent with the reduction of breast cancer metastasis in Plg^−^ mice using the PyMT mammary cancer [23]. Moreover, reduced metastatic potential was observed in a model of B16F10 melanoma cells to the brain following injection into the vasculature on Plg^−^ mice or mice  treated with the plasminogen activation inhibitor ε‐aminocaproic acid [46]. We showed that reduction of *Plgrkt*, but not *S100a10*, in PDAC tumor cells significantly reduced metastatic potential. Collectively, these findings suggest that linking plasminogen to the tumor cell surface through multiple means can promote primary growth but that specific tumor cell receptor–plasmin(ogen) interactions are required to promote metastasis.

An important translational element presented here is that pharmacological reduction of plasminogen using ASOs resulted in reduction in the growth and lung metastasis of low‐passage patient‐derived PDAC tumor cell lines in immune‐deficient mice. Plasminogen reduction resulted in significantly reduced primary tumor growth for multiple lines in both subcutaneous and orthotopic models. Unlike the KPC model, this effect was not linked to an increase in apoptosis in the orthotopic Pa03C tumors at time of sacrifice. The differences in apoptosis as measured by cleaved caspase 3 between the KPC and Pa03C models can be attributed to several factors. A potential reason for this discrepancy is the difference in the tumor microenvironment within the KPC and Pa03C model which can drive variations in the timing of the apoptotic response of tumor cells. In addition, spontaneous metastasis in the Pa03C orthotopic model was significantly reduced to the lung, but significantly increased in the liver following Plg depletion. The human cell line studies relied on pharmacological reduction of plasminogen, which was not a complete deficiency as was the case in the KPC studies. The residual plasmin activity could confer significant disease‐promoting activity. In addition, the patient‐derived tumor studies had to be conducted in immune‐deficient NSG mice that have no NK cells and compromised macrophage functions [55, 56]. NK cells are known to play a major role in suppressing metastasis, and macrophages can support PDAC tumor growth. Given that our data suggest that plasminogen supports PDAC disease progression through modifying immune cell activities, it is possible that the contribution of plasminogen to tumor progression in NSG mice could be mitigated.

## 5 Conclusions

Genetic or pharmacological depletion of plasminogen was found to suppress primary PDAC tumor growth and metastatic potential. Reduced PDAC disease was associated with increased tumor cell death, reduced infiltration of tumor‐supporting immune cells. Whereas the absence of fibrin(ogen) did not significantly influence tumor growth and had only a modest impact on metastasis, expression of *Plgrkt* or *S100a10* by the tumor cells significantly enhanced both primary tumor growth metastatic potential. Future studies will be directed toward analyses of plasmin(ogen)‐mediated modulation of immune cell function in PDAC and the single and combined contribution of plasminogen activator expression by both tumor and stromal cells to PDAC pathogenesis. Collectively, the findings presented here support the concept that tumor cell surface plasmin(ogen) activity is a significant driver of PDAC disease progression.

## Conflict of interest

ASR is an employee of Ionis Pharmaceuticals. BdL is employed by Synapse Research Institute, a not‐for‐profit member of the STAGO Diagnostic group that produces calibrated automated thrombography for thrombin generation measurements in plasma. Synapse Research Institute holds the patent on calibrated plasmin generation. All other authors declare no competing conflicts of interest.

## Author contributions

NNC, YY, AD, ML, ZW, FS, and SRA designed studies, conducted experiments, and assisted in writing the manuscript. ASR, RJP, LAM, and BdL provided necessary reagents and help with experimental design. ASW and JPL helped in designing studies and interpreting data. MLF and MJF designed the study, conducted experiments, interpreted data, and wrote the manuscript. All authors reviewed the final version of the manuscript.

## Supporting information


**Fig. S1.** Fibrin deposition is a common feature of the PDAC tumor microenvironment.
**Fig. S2.** High expression of the plasminogen receptors PLGRKT or S100A10 correlates with poor patient prognosis and reduction of *Plgrkt* or *S100A10* in KPC2 cells results in a modest but statistically significant reduction in the expression of *Cdh1*.
**Fig. S3.** Elimination of Plgrkt from the host does not alter KPC2 tumor growth in mice following orthotopic injection.
**Fig. S4.** Analysis of plasma plasmin activity, fibrinogen, and plasmin generation in mice with orthotopic Pa03C tumors.
**Fig. S5.** Pathology scoring of metastatic lesions to liver and lung harvested from Control‐ASO or Plg‐ASO treated mice with orthotopic Pa03C tumors.

## References

[1] American Cancer Society . Survival rates for pancreatic cancer. 2022. https://www.cancer.org/cancer/types/pancreatic-cancer/detection-diagnosis-staging/survival-rates.html

[2] Blom JW , Doggen CJ , Osanto S , Rosendaal FR . Malignancies, prothrombotic mutations, and the risk of venous thrombosis. JAMA. 2005;293(6):715–722. 15701913 10.1001/jama.293.6.715

[3] Horsted F , West J , Grainge MJ . Risk of venous thromboembolism in patients with cancer: a systematic review and meta‐analysis. PLoS Med. 2012;9(7):e1001275. 22859911 10.1371/journal.pmed.1001275 PMC3409130

[4] Stein PD , Beemath A , Meyers FA , Skaf E , Sanchez J , Olson RE . Incidence of venous thromboembolism in patients hospitalized with cancer. Am J Med. 2006;119(1):60–68. 16431186 10.1016/j.amjmed.2005.06.058

[5] Yang Y , Stang A , Schweickert PG , Lanman NA , Paul EN , Monia BP , et al. Thrombin signaling promotes pancreatic adenocarcinoma through PAR‐1‐dependent immune evasion. Cancer Res. 2019;79(13):3417–3430. 31048498 10.1158/0008-5472.CAN-18-3206 PMC6699516

[6] Kanda M , Matthaei H , Wu J , Hong S–M , Yu J , Borges M , et al. Presence of somatic mutations in most early‐stage pancreatic intraepithelial neoplasia. Gastroenterology. 2012;142(4):730–733.e9. 22226782 10.1053/j.gastro.2011.12.042 PMC3321090

[7] Morris JP , Wang SC , Hebrok M . KRAS, hedgehog, Wnt and the twisted developmental biology of pancreatic ductal adenocarcinoma. Nat Rev Cancer. 2010;10(10):683–695. 20814421 10.1038/nrc2899 PMC4085546

[8] Geddings JE , Mackman N . Tumor‐derived tissue factor–positive microparticles and venous thrombosis in cancer patients. Blood. 2013;122(11):1873–1880. 23798713 10.1182/blood-2013-04-460139 PMC3772497

[9] Rak J , Klement P , Yu J . Genetic determinants of cancer coagulopathy, angiogenesis and disease progression. Vnitr Lek. 2006;52:135–138. 16637463

[10] Wang J‐G , Geddings JE , Aleman MM , Cardenas JC , Chantrathammachart P , Williams JC , et al. Tumor‐derived tissue factor activates coagulation and enhances thrombosis in a mouse xenograft model of human pancreatic cancer. Blood. 2012;119(23):5543–5552. 22547577 10.1182/blood-2012-01-402156 PMC3369688

[11] Yu JL , May L , Lhotak V , Shahrzad S , Shirasawa S , Weitz JI , et al. Oncogenic events regulate tissue factor expression in colorectal cancer cells: implications for tumor progression and angiogenesis. Blood. 2005;105(4):1734–1741. 15494427 10.1182/blood-2004-05-2042

[12] Adams GN , Sharma BK , Rosenfeldt L , Frederick M , Flick MJ , Witte DP , et al. Protease‐activated receptor‐1 impedes prostate and intestinal tumor progression in mice. J Thromb Haemost. 2018;16(11):2258–2269. 30152921 10.1111/jth.14277 PMC6214773

[13] Boucher AA , Rosenfeldt L , Mureb D , Shafer J , Sharma BK , Lane A , et al. Cell type‐specific mechanisms coupling protease‐activated receptor‐1 to infectious colitis pathogenesis. J Thromb Haemost. 2020;18(1):91–103. 31539206 10.1111/jth.14641 PMC7026906

[14] Barthel D , Schindler S , Zipfel PF . Plasminogen is a complement inhibitor. J Biol Chem. 2012;287(22):18831–18842. 22451663 10.1074/jbc.M111.323287 PMC3365705

[15] Castellino FJ , Ploplis VA . Structure and function of the plasminogen/plasmin system. Thromb Haemost. 2005;93(4):647–654. 15841308 10.1160/TH04-12-0842

[16] Miyashita C , Wenzel E , Heiden M . Plasminogen: a brief introduction into its biochemistry and function. Pathophysiol Haemost Thromb. 1988;18(Suppl. 1):7–13. 10.1159/000215824 3280426

[17] Bera A , Zhao S , Cao L , Chiao PJ , Freeman JW . Oncogenic K‐Ras and loss of Smad4 mediate invasion by activating an EGFR/NF‐kappaB Axis that induces expression of MMP9 and uPA in human pancreas progenitor cells. PLoS One. 2013;8(12):e82282. 24340014 10.1371/journal.pone.0082282 PMC3855364

[18] Cavallo‐Medved D , Mai J , Dosescu J , Sameni M , Sloane BF . Caveolin‐1 mediates the expression and localization of cathepsin B, pro‐urokinase plasminogen activator and their cell‐surface receptors in human colorectal carcinoma cells. J Cell Sci. 2005;118(Pt 7):1493–1503. 15769846 10.1242/jcs.02278

[19] Kumar AA , Buckley BJ , Ranson M . The Urokinase plasminogen activation system in pancreatic cancer: prospective diagnostic and therapeutic targets. Biomolecules. 2022;12(2):152. 35204653 10.3390/biom12020152 PMC8961517

[20] Baluka D , Urbanek T , Lekstan A , Swietochowska E , Wiaderkiewicz R , Kajor M , et al. The role of the tissue plasminogen activator as a prognostic and differentiation factor in patients with pancreatic cancer and chronic pancreatitis. J Physiol Pharmacol. 2016;67(1):93–101. 27010898

[21] Gorantla B , Asuthkar S , Rao JS , Patel J , Gondi CS . Suppression of the uPAR‐uPA system retards angiogenesis, invasion, and in vivo tumor development in pancreatic cancer cells. Mol Cancer Res. 2011;9(4):377–389. 21389187 10.1158/1541-7786.MCR-10-0452

[22] Harris NLE , Vennin C , Conway JRW , Vine KL , Pinese M , Cowley MJ , et al. SerpinB2 regulates stromal remodelling and local invasion in pancreatic cancer. Oncogene. 2017;36(30):4288–4298. 28346421 10.1038/onc.2017.63 PMC5537606

[23] Bugge TH , Lund LR , Kombrinck KK , Nielsen BS , Holmbäck K , Drew AF , et al. Reduced metastasis of Polyoma virus middle T antigen‐induced mammary cancer in plasminogen‐deficient mice. Oncogene. 1998;16(24):3097–3104. 9671388 10.1038/sj.onc.1201869

[24] Miles LA , Baik N , Lighvani S , Khaldoyanidi S , Varki NM , Bai H , et al. Deficiency of plasminogen receptor, Plg‐RKT, causes defects in plasminogen binding and inflammatory macrophage recruitment in vivo. J Thromb Haemost. 2017;15(1):155–162. 27714956 10.1111/jth.13532 PMC5280214

[25] Suh TT , Holmbäck K , Jensen NJ , Daugherty CC , Small K , Simon DI , et al. Resolution of spontaneous bleeding events but failure of pregnancy in fibrinogen‐deficient mice. Genes Dev. 1995;9(16):2020–2033. 7649481 10.1101/gad.9.16.2020

[26] Prasad JM , Gorkun OV , Raghu H , Thornton S , Mullins ES , Palumbo JS , et al. Mice expressing a mutant form of fibrinogen that cannot support fibrin formation exhibit compromised antimicrobial host defense. Blood. 2015;126(17):2047–2058. 26228483 10.1182/blood-2015-04-639849 PMC4616238

[27] Mignemi NA , Yuasa M , Baker CE , Moore SN , Ihejirika RC , Oelsner WK , et al. Plasmin prevents dystrophic calcification after muscle injury. J Bone Miner Res. 2017;32(2):294–308. 27530373 10.1002/jbmr.2973

[28] Baker SK , Chen ZL , Norris EH , Revenko AS , MacLeod A , Strickland S . Blood‐derived plasminogen drives brain inflammation and plaque deposition in a mouse model of Alzheimer's disease. Proc Natl Acad Sci USA. 2018;115(41):E9687–E9696. 30254165 10.1073/pnas.1811172115 PMC6187132

[29] Miszta A , Kopec AK , Pant A , Holle LA , Byrnes JR , Lawrence DA , et al. A high‐fat diet delays plasmin generation in a thrombomodulin‐dependent manner in mice. Blood. 2020;135(19):1704–1717. 32315384 10.1182/blood.2019004267 PMC7205812

[30] Poole LG , Kopec AK , Groeneveld DJ , Pant A , Baker KS , Cline‐Fedewa HM , et al. Factor XIII cross‐links fibrin(ogen) independent of fibrin polymerization in experimental acute liver injury. Blood. 2021;137(18):2520–2531. 33569603 10.1182/blood.2020007415 PMC8109015

[31] Poole LG , Pant A , Baker KS , Kopec AK , Cline‐Fedewa HM , Iismaa SE , et al. Chronic liver injury drives non‐traditional intrahepatic fibrin(ogen) crosslinking via tissue transglutaminase. J Thromb Haemost. 2019;17(1):113–125. 30415489 10.1111/jth.14330 PMC6322974

[32] Obonai T , Fuchigami H , Furuya F , Kozuka N , Yasunaga M , Matsumura Y . Tumour imaging by the detection of fibrin clots in tumour stroma using an anti‐fibrin fab fragment. Sci Rep. 2016;6:23613. 27009516 10.1038/srep23613 PMC4806360

[33] Palumbo JS , Kombrinck KW , Drew AF , Grimes TS , Kiser JH , Degen JL , et al. Fibrinogen is an important determinant of the metastatic potential of circulating tumor cells. Blood. 2000;96(10):3302–3309. 11071621

[34] Palumbo JS , Talmage KE , Massari JV , la Jeunesse CM , Flick MJ , Kombrinck KW , et al. Platelets and fibrin(ogen) increase metastatic potential by impeding natural killer cell‐mediated elimination of tumor cells. Blood. 2005;105(1):178–185. 15367435 10.1182/blood-2004-06-2272

[35] Miles LA , Baik N , Bai H , Makarenkova HP , Kiosses WB , Krajewski S , et al. The plasminogen receptor, Plg‐RKT, is essential for mammary lobuloalveolar development and lactation. J Thromb Haemost. 2018;16(5):919–932. 29495105 10.1111/jth.13988 PMC5965281

[36] Kwon M , MacLeod TJ , Zhang Y , Waisman DM . S100A10, annexin A2, and annexin a2 heterotetramer as candidate plasminogen receptors. Front Biosci. 2005;10:300–325. 15574370 10.2741/1529

[37] Gampala S , Shah F , Lu X , Moon HR , Babb O , Umesh Ganesh N , et al. Ref‐1 redox activity alters cancer cell metabolism in pancreatic cancer: exploiting this novel finding as a potential target. J Exp Clin Cancer Res. 2021;40(1):251. 34376225 10.1186/s13046-021-02046-x PMC8353735

[38] Jones S , Zhang X , Parsons DW , Lin JC , Leary RJ , Angenendt P , et al. Core signaling pathways in human pancreatic cancers revealed by global genomic analyses. Science. 2008;321(5897):1801–1806. 18772397 10.1126/science.1164368 PMC2848990

[39] Li W , Tang Y , Song Y , Chen SH , Sisliyan N , Ni M , et al. Prognostic role of pretreatment plasma D‐dimer in patients with solid tumors: a systematic review and meta‐analysis. Cell Physiol Biochem. 2018;45(4):1663–1676. 29490291 10.1159/000487734

[40] Xu P , Wang XD , Qian JJ , Li ZN , Yao J , Xu AM . The prognostic evaluation of CA19‐9, D‐dimer and TNFAIP3/A20 in patients with pancreatic ductal adenocarcinoma. Medicine (Baltimore). 2021;100(6):e24651. 33578593 10.1097/MD.0000000000024651 PMC10545421

[41] Chen H , Li F , Zou S , Xie J , Zhang J , Deng X , et al. Preoperative plasma D‐dimer independently predicts survival in patients with pancreatic ductal adenocarcinoma undergoing radical resection. World J Surg Oncol. 2021;19(1):166. 34107980 10.1186/s12957-021-02281-8 PMC8191214

[42] Durczynski A , Skulimowski A , Hogendorf P , Szymanski D , Kumor A , Marski K , et al. The concentration of D‐dimers in portal blood positively correlates with overall survival in patients with non‐resectable pancreatic cancer. World J Surg Oncol. 2017;15(1):223. 29246148 10.1186/s12957-017-1291-4 PMC5732385

[43] Cantero D , Friess H , Deflorin J , Zimmermann A , Bründler MA , Riesle E , et al. Enhanced expression of urokinase plasminogen activator and its receptor in pancreatic carcinoma. Br J Cancer. 1997;75(3):388–395. 9020484 10.1038/bjc.1997.63 PMC2063363

[44] Aguilar S , Corominas JM , Malats N , Pereira JA , Dufresne M , Real FX , et al. Tissue plasminogen activator in murine exocrine pancreas cancer: selective expression in ductal tumors and contribution to cancer progression. Am J Pathol. 2004;165(4):1129–1139. 15466380 10.1016/S0002-9440(10)63374-3 PMC1618622

[45] Ortiz‐Zapater E , Peiró S , Roda O , Corominas JM , Aguilar S , Ampurdanés C , et al. Tissue plasminogen activator induces pancreatic cancer cell proliferation by a non‐catalytic mechanism that requires extracellular signal‐regulated kinase 1/2 activation through epidermal growth factor receptor and annexin A2. Am J Pathol. 2007;170(5):1573–1584. 17456763 10.2353/ajpath.2007.060850 PMC1854952

[46] Perides G , Zhuge Y , Lin T , Stins MF , Bronson RT , Wu JK . The fibrinolytic system facilitates tumor cell migration across the blood‐brain barrier in experimental melanoma brain metastasis. BMC Cancer. 2006;6:56. 16524486 10.1186/1471-2407-6-56 PMC1421425

[47] Palumbo JS , Talmage KE , Liu H , la Jeunesse CM , Witte DP , Degen JL . Plasminogen supports tumor growth through a fibrinogen‐dependent mechanism linked to vascular patency. Blood. 2003;102(8):2819–2827. 12829586 10.1182/blood-2003-03-0881

[48] Hald A , Eickhardt H , Maerkedahl RB , Feldborg CW , Egerod KL , Engelholm LH , et al. Plasmin‐driven fibrinolysis facilitates skin tumor growth in a gender‐dependent manner. FASEB J. 2012;26(11):4445–4457. 22815383 10.1096/fj.12-208025

[49] Silva LM , Lum AG , Tran C , Shaw MW , Gao Z , Flick MJ , et al. Plasmin‐mediated fibrinolysis enables macrophage migration in a murine model of inflammation. Blood. 2019;134(3):291–303. 31101623 10.1182/blood.2018874859 PMC6639982

[50] Phipps KD , Surette AP , O'Connell PA , Waisman DM . Plasminogen receptor S100A10 is essential for the migration of tumor‐promoting macrophages into tumor sites. Cancer Res. 2011;71(21):6676–6683. 22042827 10.1158/0008-5472.CAN-11-1748

[51] Thaler B , Baik N , Hohensinner PJ , Baumgartner J , Panzenböck A , Stojkovic S , et al. Differential expression of Plg‐RKT and its effects on migration of proinflammatory monocyte and macrophage subsets. Blood. 2019;134(6):561–567. 31221672 10.1182/blood.2018850420 PMC6688429

[52] Principe M , Ceruti P , Shih NY , Chattaragada MS , Rolla S , Conti L , et al. Targeting of surface alpha‐enolase inhibits the invasiveness of pancreatic cancer cells. Oncotarget. 2015;6(13):11098–11113. 25860938 10.18632/oncotarget.3572 PMC4484442

[53] Bydoun M , Sterea A , Liptay H , Uzans A , Huang WY , Rodrigues GJ , et al. S100A10, a novel biomarker in pancreatic ductal adenocarcinoma. Mol Oncol. 2018;12(11):1895–1916. 30009399 10.1002/1878-0261.12356 PMC6210040

[54] Lin H , Yang P , Li B , Chang Y , Chen Y , Li Y , et al. S100A10 promotes pancreatic ductal adenocarcinoma cells proliferation, migration and adhesion through JNK/LAMB3‐LAMC2 Axis. Cancers (Basel). 2022;15(1):202. 36612197 10.3390/cancers15010202 PMC9818352

[55] Shultz LD , Lyons BL , Burzenski LM , Gott B , Chen X , Chaleff S , et al. Human lymphoid and myeloid cell development in NOD/LtSz‐scid IL2R gamma null mice engrafted with mobilized human hemopoietic stem cells. J Immunol. 2005;174(10):6477–6489. 15879151 10.4049/jimmunol.174.10.6477

[56] Shultz LD , Schweitzer PA , Christianson SW , Gott B , Schweitzer IB , Tennent B , et al. Multiple defects in innate and adaptive immunologic function in NOD/LtSz‐scid mice. J Immunol. 1995;154(1):180–191. 7995938

