# Supplementary material for: Plasminogen deficiency suppresses pancreatic ductal adenocarcinoma disease progression
Source: Mol Oncol. 2023 Nov 27;18(1):113–35. doi: 10.1002/1878-0261.13552 (PMC10766200; doi:10.1002/1878-0261.13552)
Supplement: Supplementary file 1 — Fig. S1. Fibrin deposition is a common feature of the PDAC tumor microenvironment. Fig. S2. High expression of the plasminogen receptors PLGRKT or S100A10 correlates with poor patient prognosis and reduction of Plgrkt or S100A10 in KPC2 cells results in a modest but statistically significant reduction in the expression of Cdh1. Fig. S3. Elimination of Plgrkt from the host does not alter KPC2 tumor growth in mice following orthotopic injection. Fig. S4. Analysis of plasma plasmin activity, fibrinogen, and plasmin generation in mice with orthotopic Pa03C tumors. Fig. S5. Pathology scoring of metastatic lesions to liver and lung harvested from Control‐ASO or Plg‐ASO treated mice with orthotopic Pa03C tumors. [file MOL2-18-113-s001.docx]

**Supplemental Figures**

**
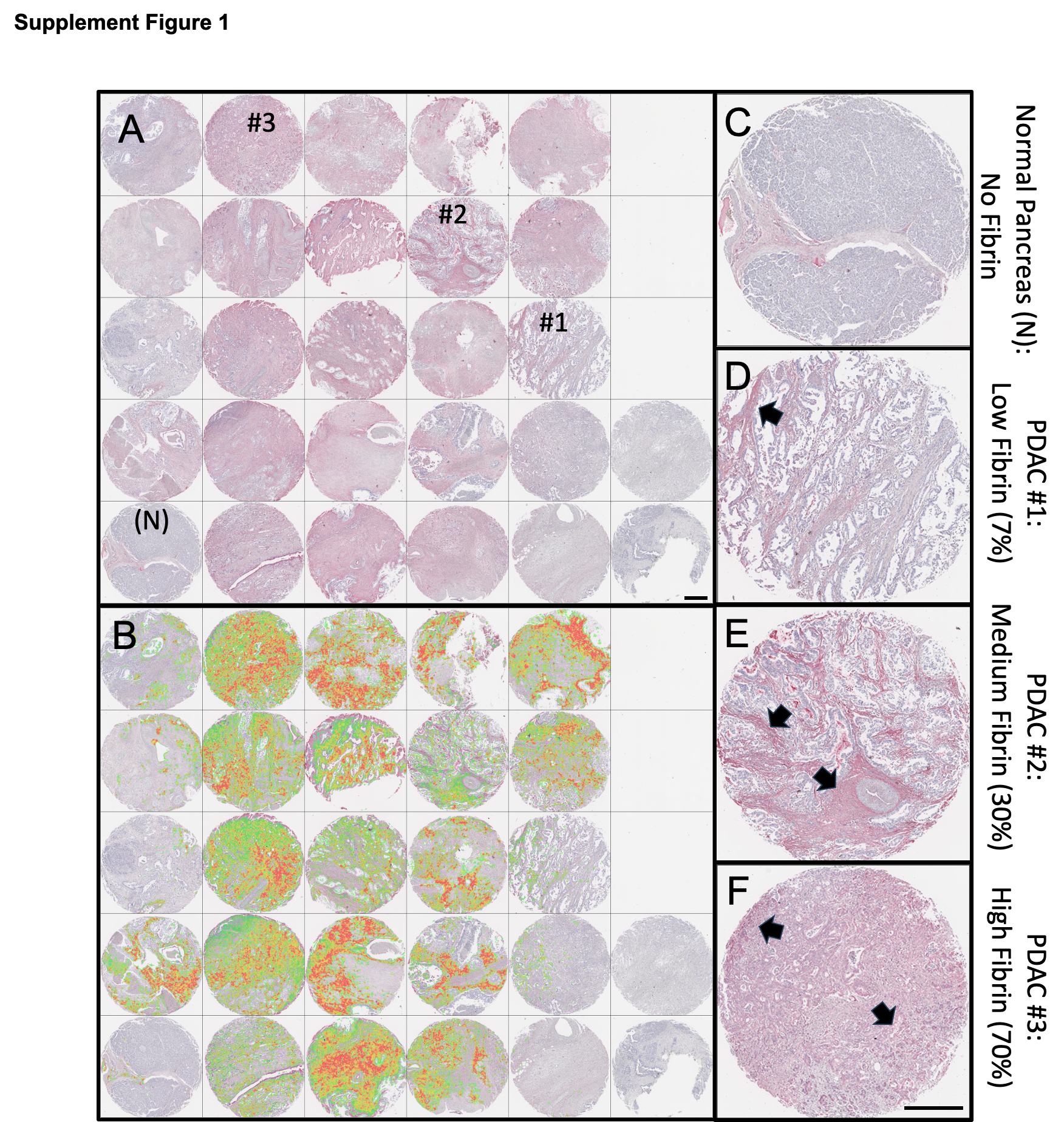
**

**Supplement Figure 1. Fibrin deposition is a common feature of the PDAC tumor microenvironment.** (A) Immunohistochemical staining for fibrin(ogen) in a human tumor microarray (TMA) of pancreatic ductal adenocarcinoma exhibited by the dark pink staining in the samples (B) HALO heat map of the TMA stained for fibrin. (C) Normal pancreas (N) with no fibrin deposition. Representative staining of PDAC tumors with varying fibrin(ogen) levels: D) PDAC #1 Low (7%), E) PDAC #2 Medium (30%) and F) PDAC #3 High (70%) levels of fibrin deposition. Scale bar= 500 μm.


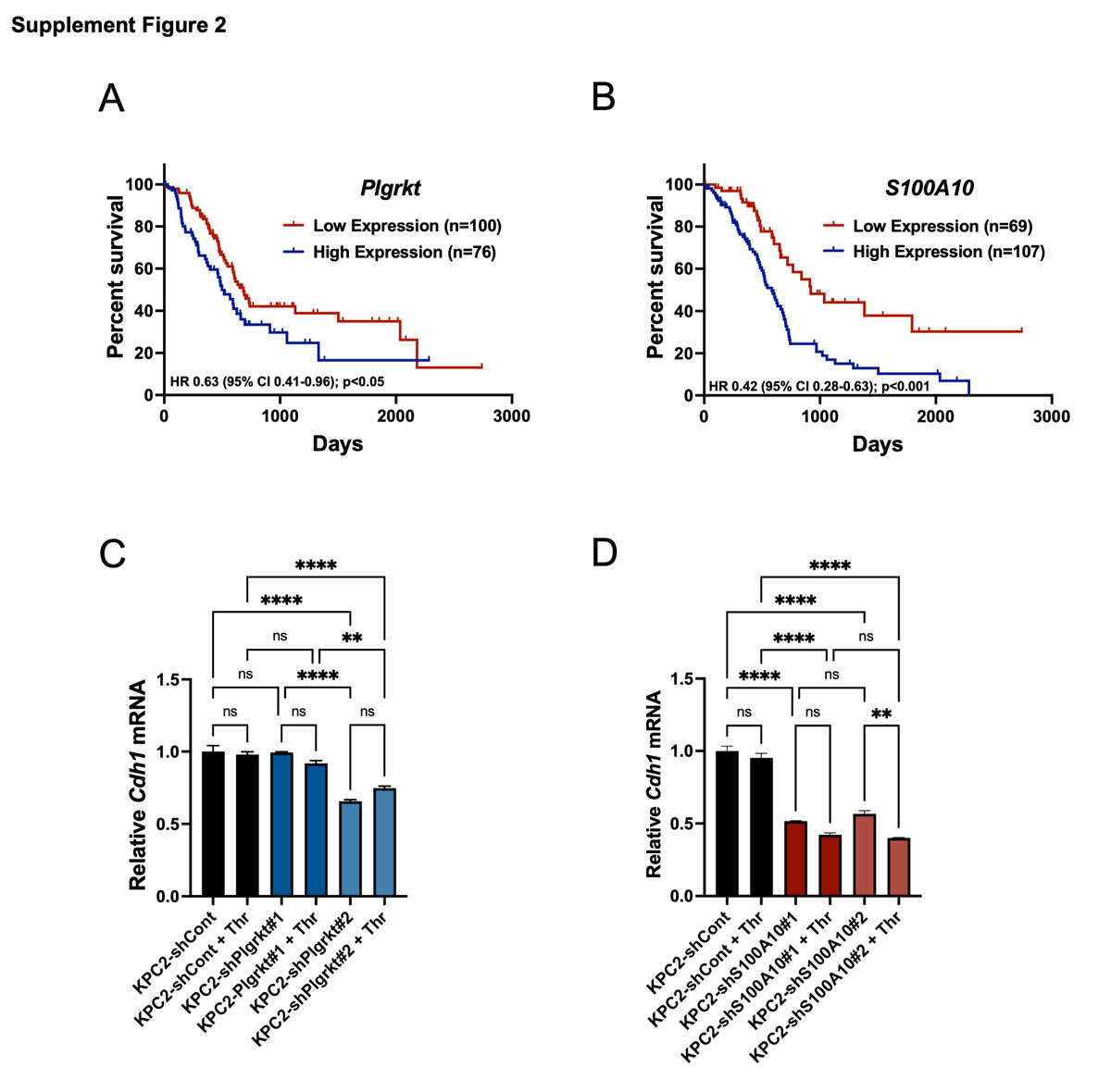


**Supplement Figure 2. High expression of the plasminogen receptors PLGRKT or S100A10 correlates with poor patient prognosis and reduction of *Plgrkt* or *S100A10* in KPC2 cells results in a modest but statistically significant reduction in the expression of *Cdh1*.** Survival analysis of PDAC patients based on tumor expression of (A) *PLGRKT* or (B) *S100A10* using TCGA data available through the Human Protein Atlas. Data is analyzed using Kaplan-Meier log rank. Quantitative RT-PCR analysis of *Cdh1* from KPC2-shControl and (C) KPC-shPlgrkt clones or (D) KPC2-shS100A10 clones (n=3 replicates per group) stimulated in vitro with or without 1 U/ml thrombin. Data are presented as mean ± SEM and analyzed by One-way ANOVA with a Tukey’s Multiple Comparison Test with **p<0.01; ****p<0.0001.


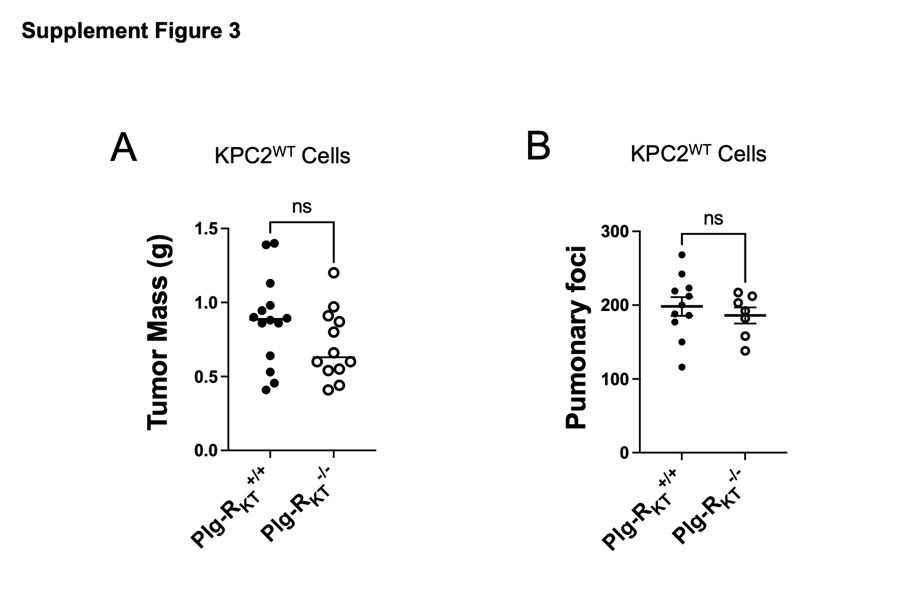


**Supplement Figure 3. Elimination of Plgrkt from the host does not alter KPC2 tumor growth in mice following orthotopic injection.** (A) Tumor mass 3 weeks after orthotopic injection of KPC2 cells into *Plgrkt^WT^* and *Plgrkt^-/-^* mice. (B) Number of surface pulmonary foci in *Plgrkt^WT^* and *Plgrkt^-/-^* mice 3 weeks following tail vein injection of KPC2 cells. Data are presented as mean ± SEM and analyzed by unpaired Mann-Whitney test.

**
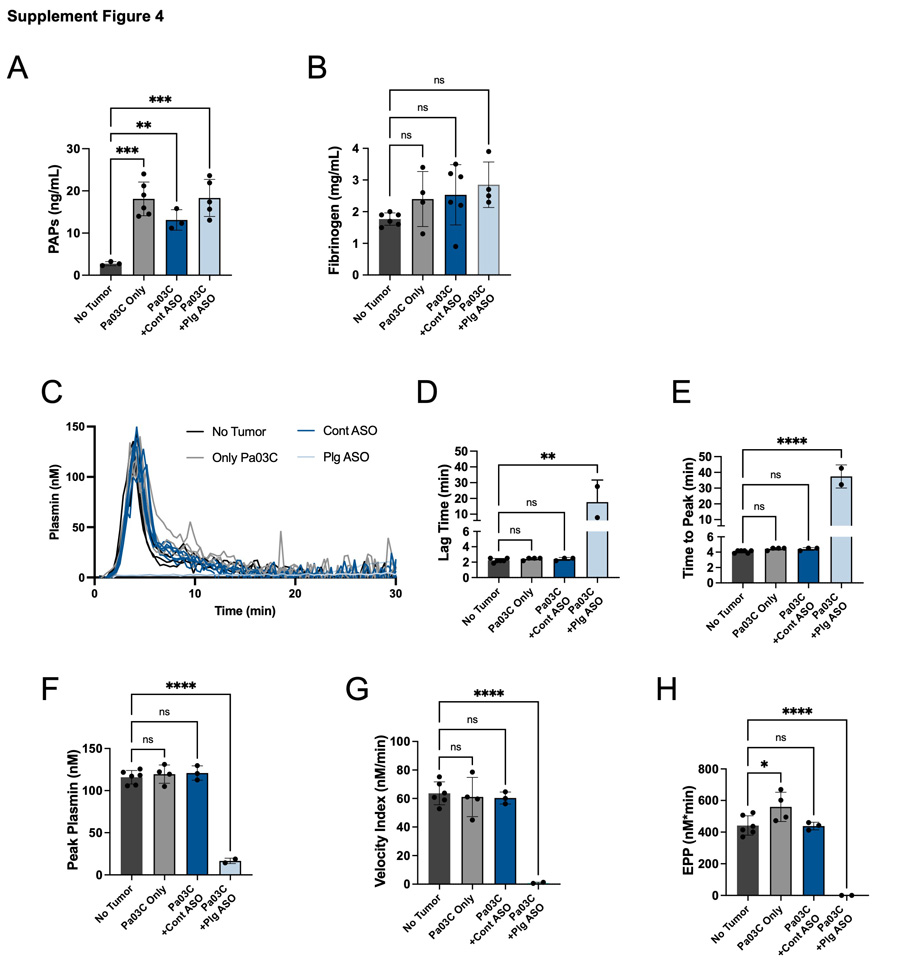
**

**Supplement Figure 4. Analysis of plasma plasmin activity, fibrinogen, and plasmin generation in mice with orthotopic Pa03C tumors.** Plasma was harvested from no tumor (naïve) mice and mice carrying Pa03C tumors that were grown for 3 weeks in mice without treatment or treated with Control ASO or Plg ASO. (A) Plasma ELISA of PAPs. (B) Plasma ELISA for fibrinogen. Plasmin generation analysis. (C) Individual curves of plasmin generation documenting the concentration of plasmin generated over time. Quantification of the individual parameters of plasmin generation, including (D) lag time, (E) time to peak, (F) peak plasmin, (G) velocity index, and (H) EPP. Data in bar graphs are expressed as the mean ± standard error of the mean. Data were analyzed by one-way ANOVA with a Dunnett’s multiple comparison test with *p<0.05, **p<0.01, ***p<0.001, ****p<0.0001.


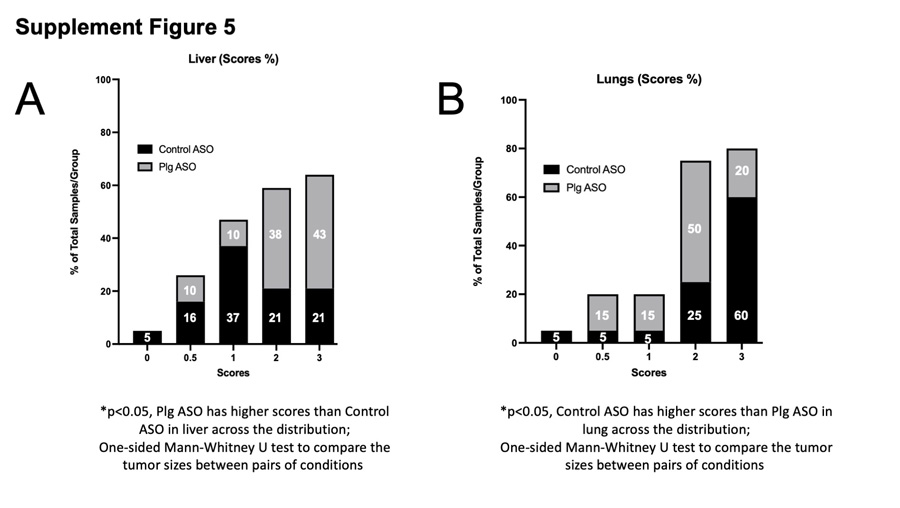


**Supplement Figure 5. Pathology scoring of metastatic lesions to liver and lung harvested from Control-ASO or Plg-ASO treated mice with orthotopic Pa03C tumors.** (A) Histology scoring of Pa03C metastases within liver tissue (Control ASO: n=20, Plg ASO: n=21). (B) Histology scoring of Pa03C metastases within lung tissue (Control ASO: n=20, Plg ASO: n=21). Size and number of lesions were compared using one-sided Mann-Whitney U test and p-values were adjusted using false discovery rate. Histological analyses of metastatic lesions are represented as the percent of total samples / group receiving scores.
